# Improving the Efficiency of Abdominal Aortic Aneurysm Wall Stress Computations

**DOI:** 10.1371/journal.pone.0101353

**Published:** 2014-07-09

**Authors:** Jaime E. Zelaya, Sevan Goenezen, Phong T. Dargon, Amir-Farzin Azarbal, Sandra Rugonyi

**Affiliations:** 1 Department of Biomedical Engineering, Oregon Health & Science University, Portland, Oregon, United States of America; 2 Department of Mechanical Engineering, Texas A & M University, College Station, Texas, United States of America; 3 Department of Surgery, Division of Vascular Surgery, Oregon Health & Science University, Portland, Oregon, United States of America; University of California Berkeley, United States of America

## Abstract

An abdominal aortic aneurysm is a pathological dilation of the abdominal aorta, which carries a high mortality rate if ruptured. The most commonly used surrogate marker of rupture risk is the maximal transverse diameter of the aneurysm. More recent studies suggest that wall stress from models of patient-specific aneurysm geometries extracted, for instance, from computed tomography images may be a more accurate predictor of rupture risk and an important factor in AAA size progression. However, quantification of wall stress is typically computationally intensive and time-consuming, mainly due to the nonlinear mechanical behavior of the abdominal aortic aneurysm walls. These difficulties have limited the potential of computational models in clinical practice. To facilitate computation of wall stresses, we propose to use a linear approach that ensures equilibrium of wall stresses in the aneurysms. This proposed linear model approach is easy to implement and eliminates the burden of nonlinear computations. To assess the accuracy of our proposed approach to compute wall stresses, results from idealized and patient-specific model simulations were compared to those obtained using conventional approaches and to those of a hypothetical, reference abdominal aortic aneurysm model. For the reference model, wall mechanical properties and the initial unloaded and unstressed configuration were assumed to be known, and the resulting wall stresses were used as reference for comparison. Our proposed linear approach accurately approximates wall stresses for varying model geometries and wall material properties. Our findings suggest that the proposed linear approach could be used as an effective, efficient, easy-to-use clinical tool to estimate patient-specific wall stresses.

## Introduction

Abdominal aortic aneurysms (AAAs) are pathological dilations of the abdominal aorta of at least 3 cm in diameter [1] [2]. If ruptured, AAAs carry a mortality rate of 90% [3], claiming approximately 15,000 American lives annually [2]. Generally, reparative surgery is performed if the AAA maximum transverse diameter measured on computed tomography (CT) scan or ultrasound imaging exceeds 5.5 cm or expands at a rate of 1 cm/year or greater [1]. But use of these criteria is concerning because AAAs smaller than 5.5 cm can rupture, and larger but stable AAAs may receive unnecessary surgery [3] [4] [5] [6] [7]. Thus, there is a need to more reliably assess the risks for AAA rupture and expansion [8].

Wall stress has been shown to be a more accurate predictor of AAA rupture and expansion than the maximum transverse diameter [5] [9] [10]. This is because tissues tear apart when wall stress exceeds a threshold stress for rupture, which depends on the tissue strength. Recent theories of growth and remodeling (G&R) [11] [12] postulate that vascular tissues grow and remodel so that homeostatic wall stresses are conserved. According to G&R theories, an increase in wall stress will result in tissue growth (e.g., increased wall thickness) and remodeling (e.g., increased collagen deposition) that lowers wall stress to homeostatic levels; likewise, an increase in wall shear stress will result in an increase in vascular diameter that will lower shear stresses to homeostatic values. These G&R mechanisms are postulated to act during AAA expansion, explaining the possible relationship between wall stress and AAA progression. Thus, wall stress has been the subject of extensive AAA biomechanical research [13] and is typically obtained using finite element analysis (FEA) [8] [14] [15] [16]. To compute wall stress, average or systolic intraluminal pressures are conventionally applied to image-derived, patient-specific geometries that are assumed to be unloaded and unstressed [17] [18]. In these models, the AAA walls are assumed to be nonlinear hyperelastic with mechanical properties measured from cadaver tissues or tissues from patients undergoing elective repair [13] [19] [20] [21]. Incorrectly assuming that imaged geometries are unloaded, however, implies that application of intraluminal pressures to the walls will result in overly distorted AAA geometries, typically with overestimated wall stress distributions [21] [22] [23] [24]. To resolve this problem, algorithms have been developed for approximating the tissue unloaded configuration from available loaded CT scan or magnetic resonance imaging (MRI) geometries [18] [22] [23] [25]. Applying intraluminal pressures to these computed unloaded geometries results in wall deformations that closely approximate the original loaded AAA geometry and more accurately predict wall stress. Although some of these promising methods have been validated, they are difficult to implement and are computationally intensive [18]. This is because computation of the undeformed unloaded configuration involves the solution of an inverse nonlinear problem. In fact, given the nonlinear properties of AAA walls, even calculation of wall stresses from a known unloaded, unstressed configuration is involved and requires extensive computations. Even when using methods to recover the unloaded geometry, limitations of current models include: residual stresses, which are characteristic of vascular tissues, are neglected; spatial changes in aneurysmal tissue properties along and across the wall are neglected; and “true” boundary conditions, including the effects of internal and external structures (thrombus and external organs), are unknown and frequently neglected or approximated. For AAA wall stresses to become a useful clinical indicator and be applicable in a clinical environment, a more efficient and robust methodology is needed for estimating wall stresses from patient-specific geometries.

In this study, we propose modeling AAAs using FEA linear models as a means of obtaining equilibrium stresses in a more robust and computationally efficient way. Because linear models assume infinitesimally small displacements and strains, the approach preserves the integrity of the imaged geometry, and the application of intraluminal pressures achieves equilibrium of forces and wall stresses directly in the patient-specific geometry. We assess the accuracy and effectiveness of our proposed approach using idealized models and patient-specific models of AAAs. We also explore the effect of employing different nonlinear wall material properties and residual stresses on wall stress computations in idealized models and compare results with those obtained from linear models.

## Problem Formulation and Equations Employed

**Figure 1:**
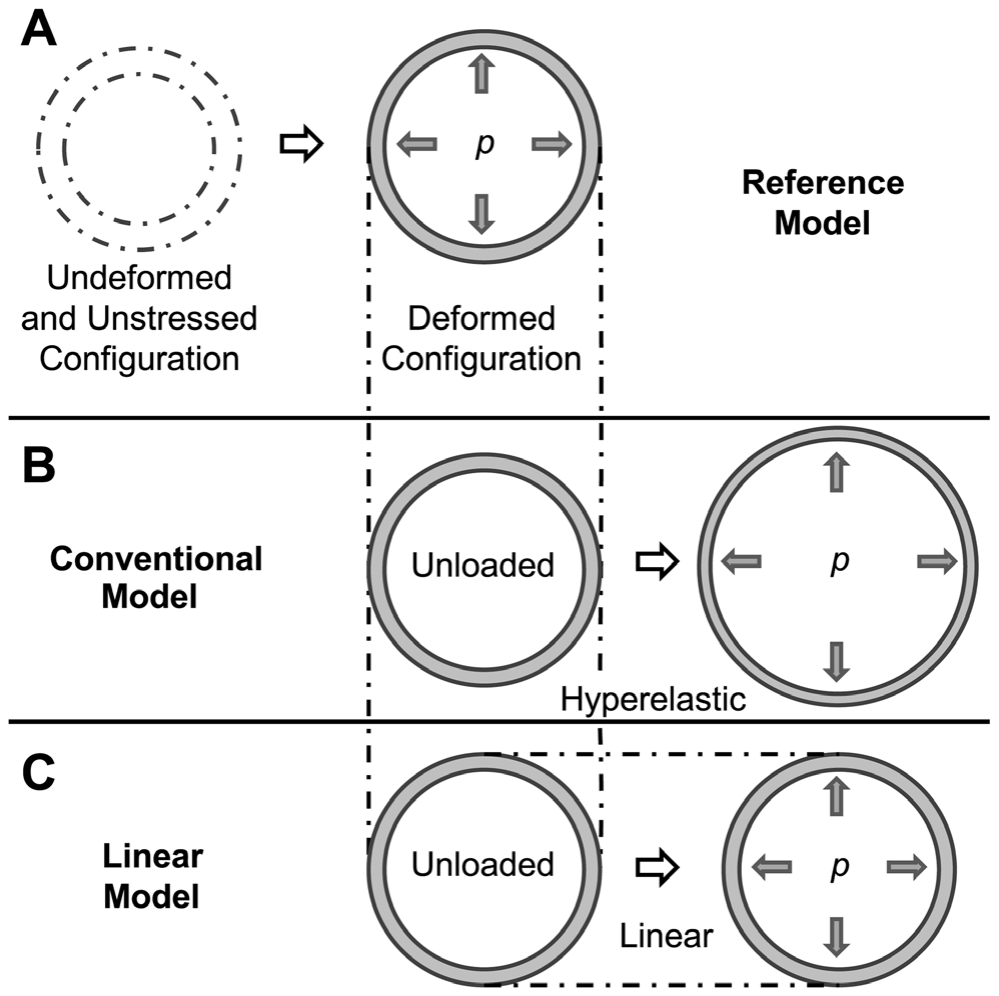
Schematic showing the AAA models employed. In all models, an internal pressure is applied to an initially unloaded, undeformed configuration. Differences between models are in the choice of the wall material properties and initial configuration employed. (A) Reference model: the walls are characterized by hyperelastic nonlinear material properties; the initial configuration, which is assumed to be known, represents the clinically unknown unloaded and unstressed wall configuration, and the deformed (loaded) configuration represents the deformed geometry that is imaged from the patient. (B) Conventional model: the walls are assumed to have hyperelastic nonlinear properties; the initial configuration is chosen as the deformed configuration obtained from the reference model (but this configuration is assumed to be unloaded and unstressed). After application of an internal pressure in the conventional model, the initial configuration further deforms into a loaded configuration. (C) Linear model: the walls are assumed to have linear elastic properties, with infinitesimally small deformations and strains; the initial configuration is chosen as the deformed configuration obtained from the reference model (as in the conventional model). Because of the assumptions made in the linear model, the initial configuration barely deforms, preserving the geometrical characteristics of imaged patient geometries.

To determine the relative accuracy of the linear approach to compute AAA wall stresses, we employed three models: a reference model, a conventional model, and our proposed linear model (see Fig. 1). The reference model (Fig. 1A) was used as a reference for wall stresses (see below for a more detailed description of the model). In the reference model, initial conditions and tissue properties are assumed to be known. The conventional model (Fig. 1B) represents the most commonly used approach to computing wall stress, in which the patient deformed configuration is used as an unloaded, unstressed initial configuration and walls are assumed to have nonlinear, hyperelastic material properties. The linear model (Fig. 1C) also uses the patient deformed configuration as an unloaded, unstressed initial configuration, but solution of the model does not change the wall geometry, and equilibrium stresses are obtained in the patient geometry. Wall stresses obtained using the linear and conventional models were compared to stresses obtained using the reference model. Comparisons were performed first using idealized models, such as straight tubular models representing the arterial wall and idealized curved axisymmetric models of the AAA. Comparisons were then extended to a subject-specific AAA geometry. For the tubular models, we further explored the effects of using different reported nonlinear tissue mechanical properties and the effects of residual stresses on wall stress. We then compared wall stresses obtained with nonlinear models to those obtained using the linear model approach.

### Model Formulation

For all models considered, we solved the equations of equilibrium
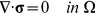
(1)with boundary conditions



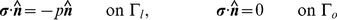
(2)where **σ** is the Cauchy stress tensor; *p* is the intraluminal blood pressure and was chosen here as the systolic pressure (0.016 N/mm^2^ = 120 mmHg) unless otherwise stated; 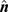 is a unit vector normal to the wall surface; Ω is the body domain in the deformed configuration; and Γ*_l_* and Γ*_o_* refer to the lumen and outer surfaces of the deformed configurations of the AAA models, respectively. Note that the choice of using systolic pressure is arbitrary and does not affect the results presented, since the hypothetical reference models are also assumed to be subjected to systolic pressure.

The reference and conventional models assumed nonlinear, hyperelastic wall material properties. Specifically, AAA walls were assumed to be almost incompressible, homogeneous, and isotropic with an energy density function *W* of the form [19] [21]

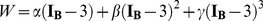
(3)where *α, β* and *γ* are coefficients that denote the properties of the tissue; **I_B_** is the first invariant of the Left Cauchy-Green tensor **B** (**I_B_** = tr**B**) with **B** = **FF** ^T^; and **F** is the deformation gradient tensor. The constitutive relations corresponding to the nonlinear material represented in Eq. 3 are described by



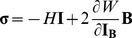
(4)where *H* is the hydrostatic pressure and **I** is the identity tensor.

The values of coefficients in Eq. 3 were determined from human tissue samples subjected to tensile tests. Material properties of AAA tissues have been measured in different studies with varying degrees of accuracy [19] [21] [26]. One of the pioneering studies, by Raghavan and Vorp [19], assumed that *W* (Eq. 3) had two terms (*γ* = 0) and fitted stress-strain results from uniaxial tissue tensile tests to find the coefficients *α* and *β* for each tissue sample. While the study found that the coefficients vary from sample to sample, it provided a population average, which is frequently used in the AAA literature to represent the material properties of AAA tissues. More recently, Polzer et al. [21] measured AAA patient tissue samples using biaxial tensile tests and fitted the resulting stress-strain curves to an energy-density function similar to that of Eq. 3 but consisting of 5 terms, i.e., *W* = *α*(**I_B_** − 3)+*β*(**I_B_** − 3)^2^+*γ*(**I_B_** − 3)^3^+*ζ*(**I_B_** − 3)^4^+*η*(**I_B_** − 3)^5^, where *ζ* and *η* are also coefficients that denote the properties of the tissue. The study found that even though *W* was assumed to be isotropic, it approximated the mechanical behavior of the tissue well. The study also found striking variations in mechanical properties among sampled tissues. Here, for comparison, we used mechanical properties obtained in the Raghavan-Vorp and Polzer studies (see Table 1, *ζ* = *η* = 0 for the two patient-specific tissue material properties selected from Polzer et al.). We extensively employed the population average mechanical properties found by Raghavan and Vorp (RV in Table 1) [19], as these properties are widely used. To assess the effects of patient-specific tissue mechanical properties, which are not known in clinical practice, we varied the values of *α* and *β* (*γ* = 0) and also used coefficients obtained by Polzer et al. from two different patient-tissue samples (P1 and P2; see Table 1) in the reference models.

**Table 1:** Coefficients of AAA tissue material properties used in this study (see Eq. 3).

Material Model	*α* (N/mm^2^)	*β* (N/mm^2^)	*γ* (N/mm^2^)	Reference
Raghavan-Vorp (RV)	0.174	1.881	0	[19]
Polzer et al. sample (P1)	0.0145	0	2.259	[21]
Polzer et al. sample (P2)	0.022	1.461	1.0	[21]

In the linear model, the wall was assumed to be an almost incompressible, linear elastic material, characterized by an arbitrary, albeit high, Young's modulus *E* (which ensures infinitesimal deformations without compromising wall stress values, see Results). Linear constitutive relations were given by



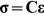
(5)where **C** is the stiffness tensor, which, for an isotropic material, depends on *E* and the Poisson's ratio *ν*, and **ε** is the infinitesimal strain tensor. Unless otherwise stated, we used *E* = 8.4×10^9^ N/mm^2^ and *ν* = 0.4999 in computations using the linear model.

Differences among the reference, conventional, and linear models were in the choice of material properties and initial configurations (see Fig. 1). In all models, the initial configuration was assumed to be unloaded and unstressed. For the reference model, the initial configuration was chosen arbitrarily and represented the unstressed and unloaded configuration of the tissue that was assumed to be known in our models (but which is unknown in clinical practice). For the conventional and linear models, however, the initial configuration was taken as the deformed configuration of the reference model. This choice of initial configuration intended to simulate the use of the loaded geometry obtained from CT scans or other imaging techniques as an unstressed, unloaded configuration, both in the conventional and linear approaches. The reference model, conversely, simulates the loading of tissues from the unknown unstressed configuration, and thus represents a more accurate model of wall stress.

Once the stress distributions were computed for all models, the wall stresses obtained from the conventional and linear models were compared to the stresses from the reference model in order to determine the degree to which conventional and linear approaches approximated reference stresses. The analysis was performed on both idealized and patient-specific models of AAAs, which are described further below. For idealized thick-wall tubular AAA models, analytical expressions exist for linear models and were derived in what follows for the hyperelastic tissue models.

### Analytical Expressions for an Axisymmetric Thick-Wall Tube under Internal Pressure

**Figure 2:**
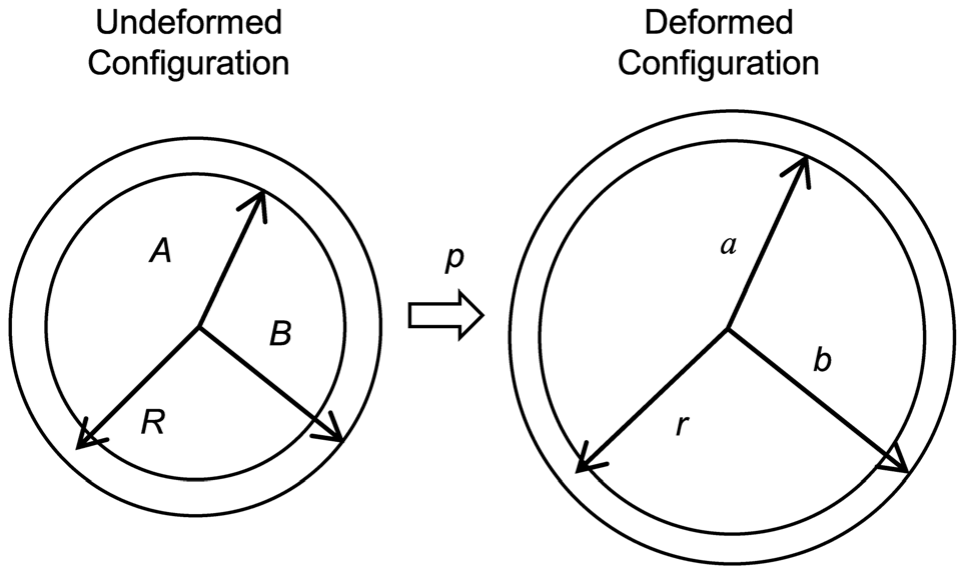
Thick-wall cylindrical model with applied internal pressure used for the derivation of analytical solutions. The undeformed configuration is assumed to be unstressed and unloaded; the deformed configuration is obtained after applying an internal pressure *p*. *A*, *B* and *R* represent the internal wall radius, external wall radius and radial coordinate, respectively, in the undeformed configuration; *a*, *b*, and *r*, represent the internal wall radius, external wall radius and radial coordinate, respectively, in the deformed configuration.

Consider an axisymmetric thick-wall tube as a simple representation of a blood vessel (see Fig. 2). Initially, the vessel is assumed to be undeformed, unloaded and unstressed with an inner radius, *A*, and outer radius, *B*. In addition, the vessel is assumed to be constrained at both ends in the longitudinal direction, and a uniform internal pressure *p* is applied on the luminal surface. Application of the internal pressure results in a deformed geometry with inner and outer radii *a* and *b*, respectively.

Employing cylindrical coordinates, the equilibrium equation (Eq. 1) for this case reduces to
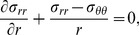
(6)where *r* is the radius of the deformed configuration and *σ_rr_* and *σ_θθ_* are the Cauchy radial and circumferential stresses, respectively [12].

The boundary conditions for the problem (Eq. 2) become



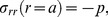
(7)

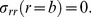
(8)


#### A. Analytical Solutions for Nonlinear Hyperelastic Tissue Model

We assume the wall properties to be incompressible and nonlinear hyperelastic with a parabolic strain energy density function *W* as proposed by Raghavan et al. [19], see Eq. 3 (with *γ* = 0) and constitutive equations given by Eq. 4.

If *λ_θθ_*, *λ_rr_*, and *λ_zz_* represent the stretch ratios in the circumferential, radial, and longitudinal directions, respectively, then for a tube under internal pressure,



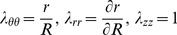
(9)where *r* and R are the radii of the deformed and initial (unstressed) configurations, respectively (see Fig. 2).

Thus, for the thick-wall tube model considered here, **F** and **B** are



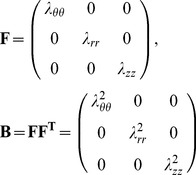
(10)

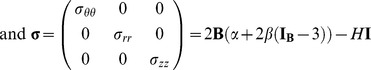
(11)


Replacing into Eq. 6 results in
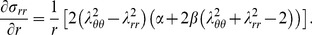
(12)


Further, for an incompressible material [12], 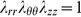
(13)and, therefore, using Eqs. 9 and 13 and solving for *r*,
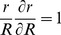
(14)

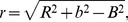
(15)




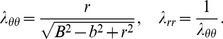
(16)


Using Eqs. 8, 12 and 16 and solving for *σ_rr_* then yields



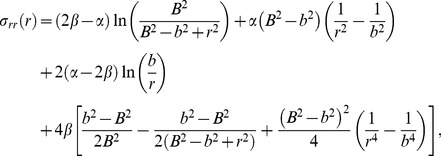
(17)and using Eq. 7,



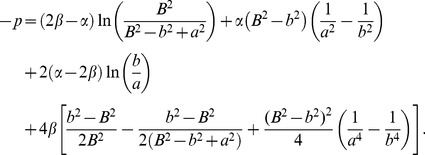
(18)


In order to solve Eq. 18 for *a* and *b*, an additional equation is needed. Since the cross-sectional areas of the undeformed and deformed configurations are equal due to incompressibility,



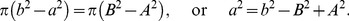
(19)


By substituting the equation for *a* in Eq. 19 into Eq. 18 and numerically solving for *b* (given *α*, *β*, *A* and *B*), the values for *b* and *a* that satisfy the boundary conditions (Eqs. 7 and 8) can be obtained.

Once *a* and *b* are computed, Eqs. 6 and 17 are used to obtain an expression for the circumferential stress,



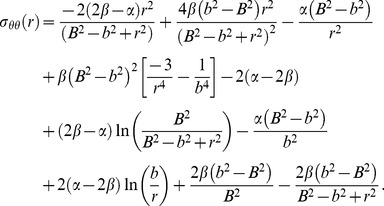
(20)


To solve for *σ_zz_*, we first find an equation for *H* using Eq. 11,



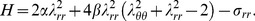
(21)


Substituting Eq. 21 into the expression for *σ_zz_* from Eq. 11 then yields,
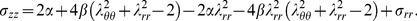
(22)


Thus the analytical solutions for wall stresses in a tubular model when tissue properties are assumed to be nonlinear hyperelastic (Eq. 3 with *γ* = 0) are given by Eqs. 17, 20 and 22, once the deformed internal and external tube radii, *a* and *b*, are calculated using Eqs. 18 and 19.

#### B. Analytical Solutions for the Linear Tissue Model

Analytical solutions for the case of a thick-wall tube under internal pressure with linear wall properties under small deformations can be found elsewhere (e.g. [27]). For completeness we included the equations together with some of the steps required in the derivation of equations. The constitutive relations for a linear elastic material, assuming infinitesimally small displacements in polar coordinates, are [27]



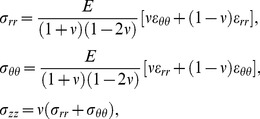
(23)where *E* is the Young's modulus and *ν* is the Poisson ratio, and *ε_rr_* and *ε_θθ_* are the radial and circumferential strains, respectively, with *ε_zz_* = 0.

For a linear axisymmetric tube with internal pressure, the strain-displacement relations in polar coordinates are
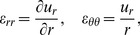
(24)where *u_r_* is the radial displacement.

Substituting Eqs. 23 and 24 into Eq. 6 results in the following differential equation:
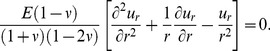
(25)


The solution of Eq. 25 is
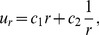
(26)where *c*_1_ and *c*_2_ are constants. Using the boundary conditions (Eqs. 7 and 8), wall stresses are obtained: 
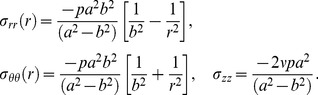
(27)


Note that, due to the assumption of small displacements, while displacements are computed, the geometry is assumed to remain unchanged (so that *a* = *A*, *b* = *B*, and *r* = *R* independent of *p*). Thus, stresses depend on *p* but not *E*.

### Effective Stress

The effective or von Mises stress is a measure of local maximum stresses that takes into account the contribution of normal stresses in addition to shear stresses and is extensively used to report stresses in the AAA literature [16] [23] [28]. However, it is worth mentioning that other measures of stress or perhaps stretch might be more relevant in determining AAA risks of expansion and of rupture [13]. Because of their wide use, however, we chose to report effective stresses in this manuscript, and for idealized AAA geometries circumferential and radial stresses were also considered. Note further that, for the purpose of this manuscript, we are not employing effective stresses as a rupture criterion, but only as a convenient way of reporting wall stresses. In cylindrical coordinates the effective stress is calculated as follows:
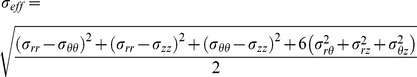
(28)


## Models of AAA

The specific geometrical models of AAA considered and strategies employed to solve for the model wall stresses are described below.

### Axisymmetric Thick-Wall Tubular Model of AAA

The arterial wall was first modeled as an axisymmetric, thick-wall, straight circular tube with applied internal pressure and no longitudinal strain. To determine how geometry affects stress distributions, wall stresses were computed from the analytical solutions using different initial configurations (i.e. initial tube dimensions, see Fig. 2). These initial configuration geometries were employed in the reference model, and the resulting deformed configuration of the reference model was used as the initial unloaded configuration in the conventional and linear models (see Fig. 1). Additionally, an array of material property values was tested to assess the effect of tissue mechanical properties on stress distributions. To simulate the clinical situation in which wall tissue properties are not known, we allowed the values of *α* and *β* to vary in the reference model (*γ* = 0), while using RV population average values in the conventional model (see Table 1) and a constant elasticity (*E* = 8.4×10^9^ N/mm^2^) in the linear model. Wall stresses from the linear and conventional models were then compared to corresponding reference stresses.

We also studied the effect of using different AAA tissue properties (RV, P1, and P2, see Table 1) on wall stresses. Since analytical expressions were not available for all material properties, we employed FEA implemented in ADINA (v8.8.3, ADINA R & D, Inc., Watertown, MA) to solve for wall stresses in an idealized 2D axisymmetric tubular model. To discretize the 2D geometry, we used optimal 9/3 axisymmetric elements, which are quadrilateral mixed displacement/pressure based elements (with 9 displacement degrees of freedom and 3 pressure degrees of freedom) that satisfy the inf-sup condition, ensuring numerical stability when solving problems involving incompressible or almost incompressible media such as the AAA wall tissue [29]. In our models, we used six elements spanning the thickness of the wall. Simulations were performed such that the deformed configuration of the hyperelastic models, used here as reference models, was the same regardless of the specific nonlinear material property employed. This deformed configuration, further, was used as the initial unloaded geometry for the linear model. Wall stresses obtained from the reference models were then compared to stresses from the linear model.

For the nonlinear FEA models presented here and throughout the study, the convergence criterion for equilibrium iterations was specified by energy. The convergence ratio for out-of-balance energy was set to a tolerance value of 0.001. The nonlinear iteration scheme used was the full Newton method, and the maximum number of iterations implemented for every time step was set to 15. Convergence was achieved for non-linear models using 15 to 60 time steps.

**Figure 3:**
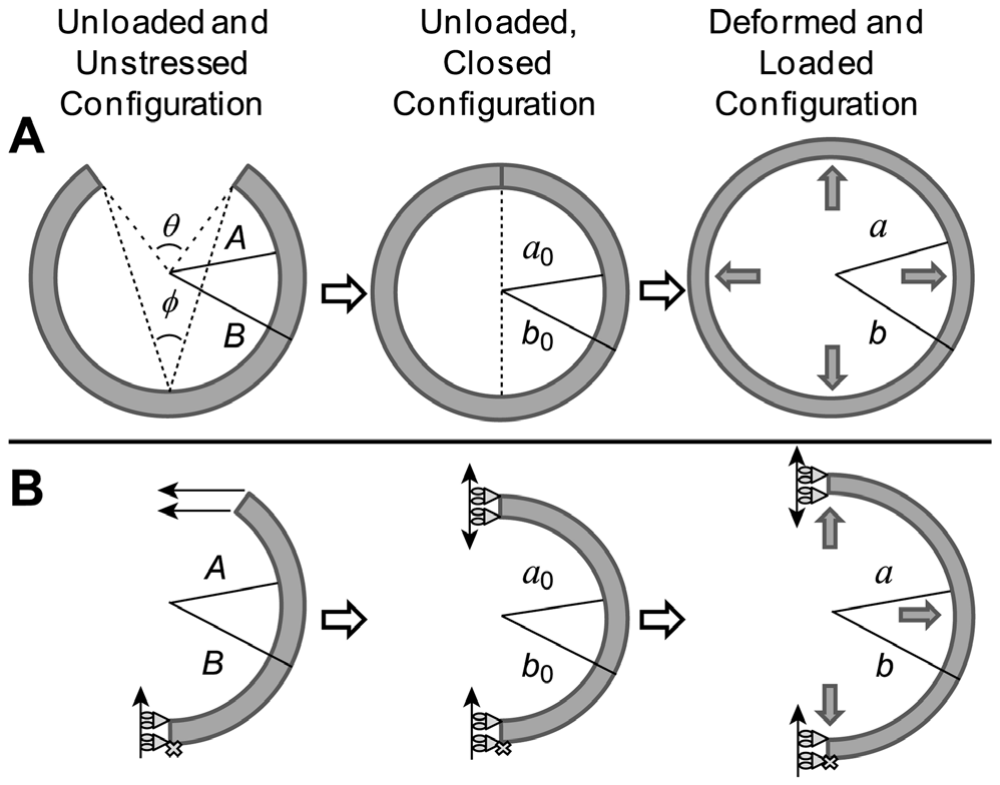
Modeling of residual stresses in a tubular vessel. (A) Schematics of the physical tissue configurations: an initial unloaded, unstressed and undeformed circular section (with radii *A* and *B*) is closed, which generates residual stresses in the unloaded configuration. This closed configuration (with radii *a_0_* and *b_0_*) is loaded to generate the final deformed and loaded configuration (radii *a* and *b*) that represents arterial tissues under load. The open sector schematics also show the definition of the opening angle, *φ*, in relation to the angle *θ*. (B) Schematics of the FEA model implemented to compute residual stresses and their effects on the loaded configuration. We first modeled an unstressed, unloaded and undeformed open sector. We then imposed a horizontal displacement on the open end of the sector to close the segment and generate residual stresses. The closed sector was then loaded with an internal pressure to compute the loaded configuration and resulting wall stresses.

We further explored the effect of residual stresses on wall stress distributions. Residual stresses are the stresses that remain on a vascular wall after loads imposed on the tissue have been removed. They manifest in blood vessels as a shrinkage in the axial length when vessel segments are cut longitudinally (axial stresses) and as an opening of the unloaded circular cross-section, characterized by an opening angle [30], when vessel segments are cut radially (circumferential stresses). To model circumferential residual stresses in tubular models of AAA, we started from a 2D open sector in the initial configuration (see Fig. 3A). The dimensions of the sector were determined so that the closed unloaded configuration was the same, independent of opening angle. The open sector was modeled as a plane strain 2D problem in ADINA, and symmetry was considered by modeling half of the sector (see Fig. 3B). The open sector was then closed by imposing a displacement in the direction of closure on one end of the sector. Note that this way of modeling residual stresses cannot be implemented in patient-specific models of the AAA. Once the 2D segment was closed, an internal pressure (*p* = 0.016 N/mm^2^) was imposed to obtain the distribution of wall stresses. The obtained deformed configuration served as the initial, unloaded, unstressed geometry of the linear model, and wall stresses obtained with the linear model and nonlinear models with varying residual stresses were then compared. Different hyperelastic material properties (RV, P1 and P2; see Table 1) were employed to determine the effect of tissue mechanical properties on residual and loaded wall stresses. The geometry was discretized using mixed 9/3 elements, and convergence of results was achieved using 180 elements, with 3 elements spanning the wall thickness.

### Idealized AAA Models with Non-Uniform Wall Thickness

We implemented idealized geometrical models of AAAs with non-uniform wall thickness to explore the effect of varying thickness on wall stress computations. To this end, we started using a thick-wall tubular model of the AAA with no residual stresses, in which wall thickness varied longitudinally. We also simulated a model in which the wall thickness varied circumferentially, assuming plane strain conditions. Analytical solutions were not available for these cases; therefore, the analysis was performed using FEA in ADINA. Mixed 9/3 elements were used to discretize the geometries. RV material properties were used here for the reference and conventional models. Reference, conventional, and linear models were simulated with the described non-uniform wall thickness. Wall stresses were then compared to establish the accuracy of the linear model in accounting for changes in wall thickness.

### Idealized Curved Axisymmetric Model of AAA

To assess the effect of wall curvature on the stresses, the arterial wall was modeled as a curved axisymmetric structure. The outer wall radius *B* of the 2D axisymmetric initial configuration was specified by the following equation:



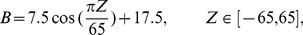
(29)where Z is the height, and *B* and Z are in mm. The height was chosen to be 130 mm [31]. The maximum diameter was 50 mm, and the wall thickness was 1.5 mm, the reported median thickness [14] [19]. The model was constrained at both ends in the longitudinal direction but was allowed to move and deform freely in the radial direction. The analysis was performed using FEA in ADINA, with the AAA wall discretized using 9/3 mixed elements.

For these models, we further incorporated an intraluminal thrombus (ILT) in our FEA simulations and compared results with and without the thrombus. When the ILT was modeled, the lumen radius, *L*, was specified by,



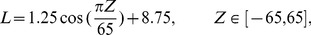
(30)and the ends of the thrombus were fixed in the longitudinal direction only. The thrombus was also discretized using mixed 9/3 elements.

Like the AAA wall, the thrombus was treated as a nonlinear, homogeneous, isotropic, incompressible material but with the following energy-density function:
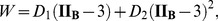
(31)where *D*_1_ and *D*_2_ are coefficients [32], and **II_B_** is the second invariant of the Left Cauchy-Green deformation tensor **B** (**II_B_** = 0.5 [(tr**B**)^2^-tr(**B**)^2^]). The ranges of measured values for *D*_1_ and *D*_2_ (95^th^ percentile confidence intervals) obtained from patients undergoing elective repair were as follows: *D*_1_ = 0.0199–0.036 N/mm^2^ and *D*_2_ = 0.0216–0.0356 N/mm^2^ [32]. In general, the thrombus is more compliant than the tissue wall. The stiffness ratio between the wall and the ILT, which we refer to as the material property ratio (MPR), was computed from the nonlinear models using the ratio of the wall coefficient *α* (Eq. 3) and the intraluminal thrombus coefficient *D*_1_ (Eq. 31), i.e., (*α*/*D*_1_). Because the MPR determines differences in stresses between the wall and ILT, the linear models that included a thrombus were implemented assuming an MPR between the elastic moduli *E* of the wall and ILT. MPR was first set at 6.7, which is the ratio of the population average values of the wall and ILT, i.e., *α = *0.174 N/mm^2^/*D*_1_ = 0.026 N/mm^2^. In order to determine how implementation of different MPRs affected the distribution of stresses, we varied MPR in our computations and compared computed wall stresses. The AAA wall material properties were modeled using the energy-density function *W* proposed by Raghavan and Vorp [19], Eq. 3 with *γ* = 0. Varying MPRs were obtained by changing the coefficient of the thrombus *D*_1_ and the coefficient *α*, for the wall. Values of MPR considered were 4, 6.7, and 10.25. An MPR of 4 was achieved by modeling a weak wall stiffness (*α* = 0.144 N/mm^2^, *β* = 1.152 N/mm^2^) and relatively stiff thrombus (*D*_1_ = 0.036 N/mm^2^, *D*_2_ = 0.0356 N/mm^2^). Conversely, an MPR of 10.25 was achieved by modeling a relatively stiff wall (*α* = 0.204 N/mm^2^, *β* = 2.61 N/mm^2^) and weak thrombus (*D*_1_ = 0.0199 N/mm^2^, *D*_2_ = 0.0216 N/mm^2^) [19] [32]. For the linear model, the wall elasticity modulus was set at a value of *E* = 8.4×10^9^ N/mm^2^, and different MPR values were generated by varying the elasticity modulus of the thrombus. For the models excluding and including the ILT, convergence was achieved with 390 and 1,690 elements, respectively, with 3 and 10 elements spanning the thickness of the wall and thrombus, respectively.

### Subject-Specific Model of AAA

To assess the effect of AAA geometry on wall stress distributions and the degree to which the conventional and linear models correctly capture these distributions, a patient-specific model was implemented. The initial configuration of the patient-specific AAA had no ILT and was extracted from contrast-enhanced spiral CT scan images of a de-identified patient. The images for this retrospective study were provided by the Oregon Health & Science University Department of Vascular Surgery following OHSU Institutional Review Board (IRB) protocols. Patient consent has been waived by the OHSU IRB, as this retrospective study constituted a minimal risk chart review. This study was approved by the OHSU IRB.

Extraction of the AAA geometry from CT scan images was achieved by using a semi-automated custom-made segmentation program. The segmentation of the vessel lumen commenced below the aortic-renal intersection and ended at the aortic-iliac bifurcation. The first contour was manually traced around the contrast-enhanced lumen of the image. Subsequent contours were automatically obtained along the AAA midline and around the contrasted lumen. Automatically segmented contours were examined and corrected manually when needed. Smoothing algorithms were applied to reduce surface-extraction noise. Contours were then “stacked” to generate the three-dimensional AAA lumen geometry in the form of a surface mesh. The mesh coordinates were imported into a custom-made MATLAB (vR2010b, MathWorks, Inc., Natick, MA) program written to uniformly displace each vertex 1.5 mm radially outward to generate the AAA outer wall geometry. This geometry was used as the unloaded configuration of the reference model. The deformed configuration obtained from the reference model, assuming RV material properties, was subsequently employed as the unloaded configuration for the conventional and linear models, as done with the other geometrical AAA models described before. The patient-specific AAA geometry was imported into ADINA and discretized using 3D, 27/4 hexahedral mixed elements with 3 elements spanning the wall thickness. The 27/4 mixed displacement/pressure elements are the 3D counterparts of the 9/3 mixed elements (with 27 displacement degrees of freedom and 4 pressure degrees of freedom) and also satisfy the inf-sup condition [29]. The ends of the model were constrained in the longitudinal direction, and selected end-nodes were constrained in all directions to prevent rigid body motion. Convergence of results was achieved using 4,800 elements.

### Wall Stress Comparisons

Wall stresses obtained from the conventional and linear models were compared to stresses obtained from reference models. Point-by-point differences in stresses were computed as follows:
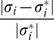
(32)where *σ_i_* is the stress of interest (conventional or linear model; circumferential, radial or effective stress) and σ*_i_ is the corresponding stress in the reference model. Eq. 32 was also used to calculate differences in maximum effective stresses with respect to those in the reference model. For models solved using FEA, stress differences with respect to the reference model were plotted for the whole model and over the wall thickness. These analyses allowed for an objective comparison of wall stress.

To facilitate comparisons of solutions over a range of tissue mechanical properties (characterized by Eq. 3 with *α* and *β*; *γ* = 0), differences in stress were integrated over the normalized thickness and normalized to the reference stress integral, 
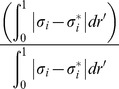
(33)where *dr'* is the normalized thickness differential. For convenience, this calculation was employed only for the axisymmetric tubular models using analytical solutions of stresses.

## Results

### Convergence of Linear Model to Equilibrium Stresses

To ensure that the linear model with applied internal pressures achieved the same equilibrium stresses independent of the Young's modulus, *E*, employed, a convergence study was first performed. Analytically, for the axisymmetric tubular model, wall stresses depended on the radius and wall thickness of the initial, undeformed configuration and the applied internal pressure. Further, wall stresses depended on wall material properties in the case of hyperelastic tissues (see Eqs. 17, 20 and 22) but were independent of wall mechanical properties when tissues were assumed to be linear and elastic with infinitesimally small deformations and strains (see Eqs. 27). As expected, when the linear axisymmetric models were implemented using FEA (assuming small displacements and strains), wall stresses did not vary significantly (<0.1%) as *E* was increased from 1 to 10^10^ N/mm^2^. Similarly, wall stresses did not vary significantly with varying values of *E* (<0.2%) in linear idealized and patient-specific models of an AAA. Estimation of equilibrium stresses using the linear model was therefore effectively independent of the *E* employed. We chose an arbitrary high Young's modulus (*E* = 8.4×10^9^ N/mm^2^) to use in our linear models. In applying this choice of elasticity modulus, the wall displacements computed for the idealized and patient-specific AAA models were negligible (<1.3×10^−9^ mm).

### Axisymmetric Tubular Model of an AAA with Parabolic Energy-Density Function

**Figure 4:**
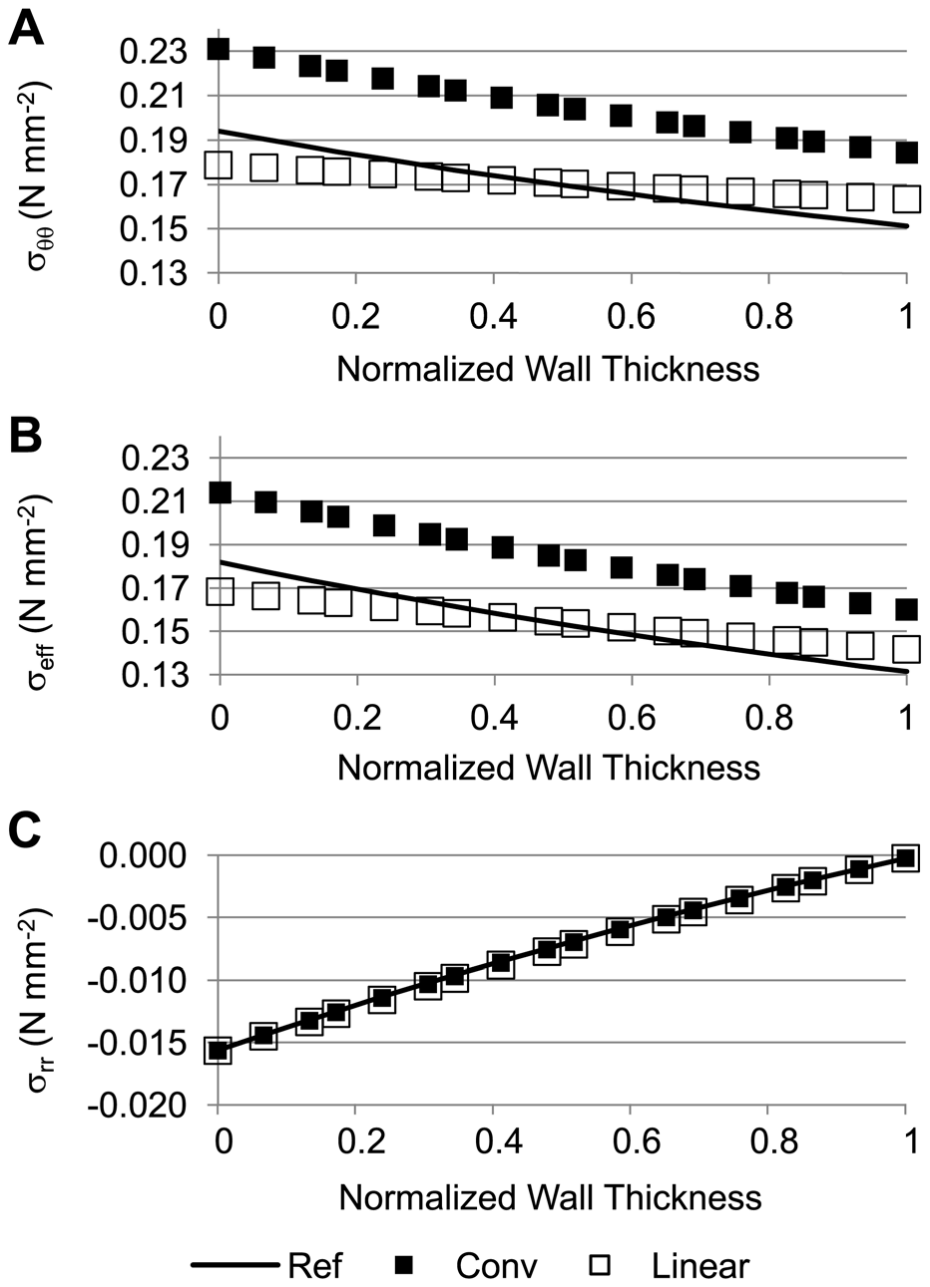
Stress comparisons among the reference, conventional, and linear models of an axisymmetric thick-wall tubular geometry. (A) Circumferential wall stress; (B) effective wall stress; and (C) radial wall stress distributions are plotted across the normalized wall thickness. For the reference model, the inner and outer radii in the deformed configuration were 14.8 mm and 16.1 mm, respectively; for the conventional model, the inner and outer radii in the deformed configuration were 16.27 mm and 17.53 mm, respectively; applied internal pressure, *p* = 0.016 N/mm^2^ (120 mmHg); RV material properties were used for both the reference and conventional models.

Wall stress versus normalized wall thickness plots were initially generated for the axisymmetric tubular model to determine how the wall stresses of the linear and conventional models compared to those of the reference model (see Fig. 4). Here, conventional and reference models used the RV material properties (see Table 1). Compared to the radial (*σ_rr_*) and axial stresses (*σ_zz_*), the circumferential stresses (*σ_θθ_*) had larger magnitude values, contributing the greatest weight to the calculation of effective stresses (see Eq. 28). Values of *σ_θθ_* computed using the linear model were closer to those obtained from the reference model than values obtained using the conventional model. A similar finding was observed for the effective stress. On the other hand, *σ_rr_* was almost the same throughout the wall thickness for all models, representing the effect of the pressure boundary conditions on radial stresses.

For a thick-wall tubular model under equilibrium, the following condition is satisfied, 
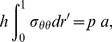
(34)where *h* and *a* are the wall thickness and lumen radius of the deformed configuration, respectively; *dr'* is the normalized thickness differential; and *p* the applied internal pressure (see Fig. 2). The integral of *σ_θθ_* over the thickness (left hand side of Eq. 34), was about 0.24 N/mm for both the linear and reference models. This was expected since the deformed wall configuration was practically identical for both models, and the boundary conditions were the same. The integral, however, was larger for the conventional model (0.26 N/mm), reflecting the additional radial expansion of the wall under the conventional approach.

**Figure 5:**
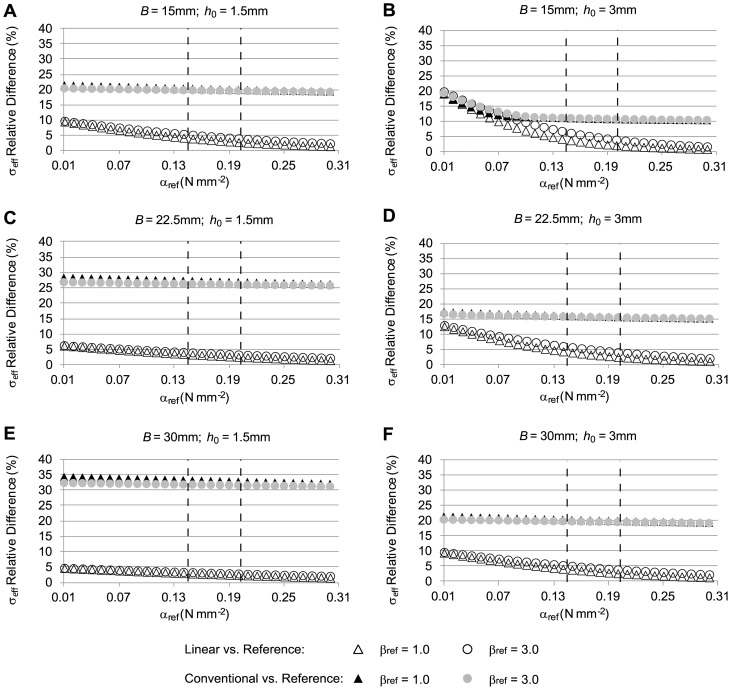
Relative differences in effective wall stress distributions for the case of a tubular arterial model. The figure shows differences of effective wall stress distributions obtained from the conventional and linear models, with respect to the stress distributions from the reference model, computed using Eq. 33. To simulate the clinical situation where the material properties of the AAA are unknown, the material constants *α* and *β* (*γ* = 0) were varied in the reference model (*α_ref_* and *β_ref_* reported values), whereas population average *α* and *β* (*α* = 0.174 N/mm^2^, *β* = 1.881 N/mm^2^; RV material properties) were used in the conventional model, and constant elasticity (*E* = 8.4×10^9^ N/mm^2^) was used in the linear model. Further, the initial geometry was varied to represent different aneurysm sizes and wall thicknesses. In all cases, applied internal pressure was 0.016 N/mm^2^ (120 mmHg). Reported geometrical model external radius, *B*, and wall thickness, *h_0_*, correspond to the initial configuration of the reference model. The dashed lines indicate the physiological range of the material property values for *α_ref_*.

To assess how closely the linear and conventional approaches approximated reference stresses under different conditions, computations were performed for a range of *α* and *β* values (*γ* = 0) and different initial geometries (see Fig. 5). To effectively compare and visualize stress differences (with respect to reference stresses) as a function of the parameters *α* and *β* in the reference model, we used Eq. 33 so that each case (linear, conventional) was represented by one value, which we chose to report as a percent stress difference. We found that, irrespective of the tissue properties used in the reference model, stresses obtained using the linear model were closer to the stresses in the reference model than were the stresses obtained using the conventional model (see Fig. 5). Differences with respect to reference stresses decreased for both the linear and conventional models as *α* increased. Increasing *β*, however, had only a small effect on stress differences. Increasing the model external radius, *B*, while keeping a constant wall thickness, *h_0_*, resulted in increased stress differences between the conventional and reference models and decreased differences between linear and reference models (compare Figs. 5A, 5C and 5E; and 5B, 5D and 5F). Both *B* and *h_0_* correspond to the initial configuration of the reference model. Increasing *h_0_* while keeping *B* constant resulted in decreased stress differences between the conventional and reference models but an increased difference between the linear and reference models (compare Figs. 5A and 5B; 5C and 5D; 5E and 5F). In general, however, the linear model approximated reference stresses better than the conventional model.

**Figure 6:**
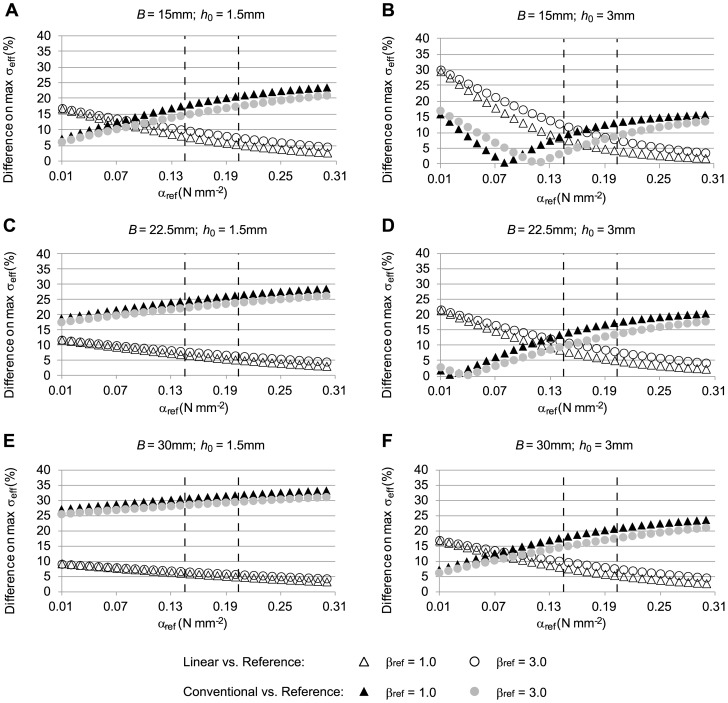
Relative differences in maximum effective wall stress distributions for a tubular arterial model. Models simulated are the same as for Fig. 5, but differences in maximal wall stresses with respect to reference stresses, computed using Eq. 32, are reported instead. The reported material coefficients *α_ref_* and *β_ref_* correspond to those of the reference model. RV material properties (*α* = 0.174 N/mm^2^, *β* = 1.881 N/mm^2^) were used in the conventional model, and a constant elasticity (*E* = 8.4×10^9^ N/mm^2^) was used in the linear model. Applied internal pressure was 0.016 N/mm^2^ (120 mmHg). The initial geometry was varied to represent different aneurysm sizes and wall thicknesses. The dashed lines indicate the physiological range of the material property values for *α_ref._*

To determine how well the maximum effective stress is approximated by the linear and conventional approaches, we examined our previous results (Fig. 5) but reported differences in maximum effective stress (see Fig. 6). Maximum stresses were generally overestimated in the conventional model and underestimated in the linear model (see Fig. 4) when tissue properties for the reference model were within physiological range. We found that in most cases, the linear model approximated the maximum stress better than the conventional model within the physiological range of *α* and *β*, and the difference gap between linear and conventional models increased with increasing diameter and decreased with increasing thickness.

Increasing the applied internal pressure in the models from 0.016 N/mm^2^ to 0.027 N/mm^2^ (120 mmHg to 200 mmHg) increased the magnitude of the differences in wall stresses for the conventional model but not for the linear model (see Figs. S1 and S2). After increasing internal pressure, the linear model provided the better approximation of maximum effective stresses within the physiological range of tissue mechanical properties.

### Idealized AAA Models with Non-Uniform Wall Thickness

**Figure 7:**
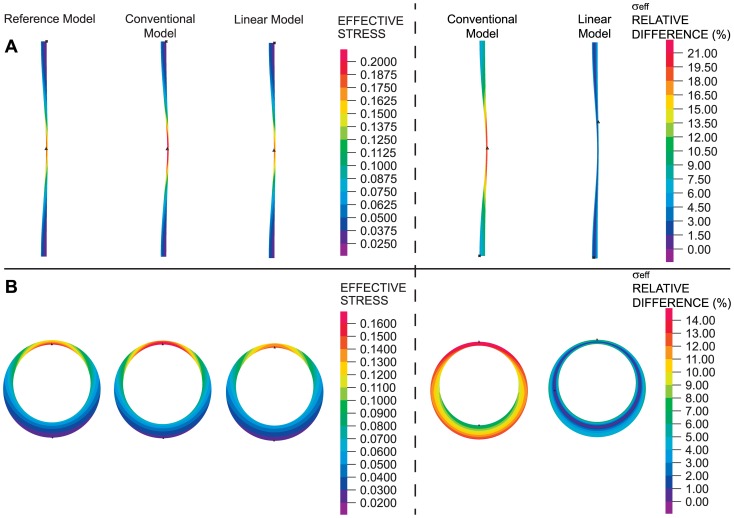
Effect of variable wall thickness on wall stress distributions. (A) Axisymmetric model with longitudinally-varying wall thickness. (B) Plane strain model with circumferentially-varying wall thickness. In all cases, an internal pressure of 0.016 N/mm^2^ (120 mmHg) was applied. For both geometries considered, effective wall stresses (in units of N/mm^2^) are shown as computed using reference, conventional and linear models (left). Differences in the effective wall stress with respect to reference wall stresses for the linear and conventional approaches are also shown (right).

To explore whether the linear model could be used to effectively study the effect of varying wall thickness, wall stresses were computed on idealized models of non-uniform wall thickness (see Fig. 7). RV material properties were used for the reference and conventional models. While the wall stresses obtained from the reference, conventional and linear models were similar, the stresses obtained using the linear model were closer approximations of the reference stresses. The linear approach could therefore be used for estimating wall stresses and studying the effects of wall thickness.

### Idealized and Subject-Specific AAA Models

**Figure 8:**
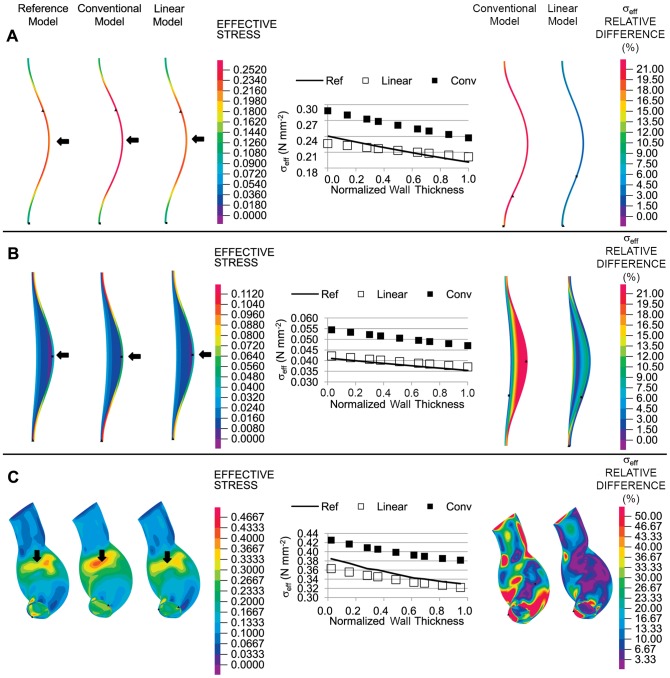
Effective wall stress distributions in different geometrical models of AAA. (A) Idealized bended-tubular axisymmetric model. (B) Idealized axisymmetric model of AAA with inclusion of thrombus. (C) Patient-specific model. In all cases, an internal pressure of 0.016 N/mm^2^ (120 mmHg) was applied. For the geometries considered, effective wall stresses (in units of N/mm^2^) are shown as computed using reference, conventional and linear models (left). Plots of the effective wall stress with respect to the normalized thickness in the regions indicated by the black arrows are shown (middle). Differences in the effective wall stress with respect to reference wall stresses for the linear and conventional approaches are also shown (right).

To determine how the wall stresses of the linear and conventional models compared to the wall stresses from the reference model when curvature was considered, we used an idealized axisymmetric model of an AAA in which the walls were curved and an internal pressure was applied to the inner wall (see Fig. 8A). RV tissue mechanical properties were used for the reference and conventional models. Effective wall stresses computed using the reference, conventional or linear models were similar, with larger stresses found in the wall region with greater curvature. Plots of wall stresses across the wall thickness and differences in effective stresses with respect to the reference configuration computed using Eq. 32 further revealed that the linear model approximated the reference stresses better than the conventional model.

**Figure 9:**
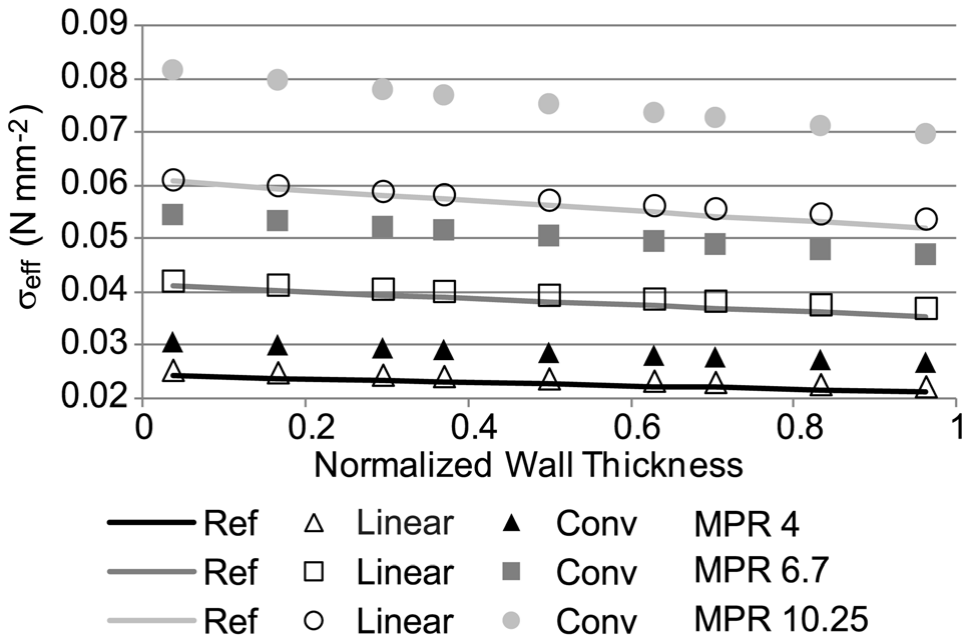
Effective stress distributions versus normalized wall thickness for the idealized AAA model with thrombus. Simulations were performed assuming a wall/thrombus material property ratio (MPR) of 4, 6.7 and 10.25, in the reference, conventional and linear models. In all cases, an internal pressure of 0.016 N/mm^2^ (120 mmHg) was applied. Ref: reference model, Conv: conventional model.

We then incorporated an intraluminal thrombus (ILT) to the idealized AAA model to assess its effect on wall stress and determine the degree to which the linear and conventional approaches approximate the reference stresses in the presence of the ILT (see Fig. 8B). To this end, we used average values of material properties for both the wall (RV properties) and thrombus in the reference and conventional models, and we used the average MPR for the linear model. We found that effective stresses that were computed using the reference model were better approximated by the linear model than by the conventional model. We then varied the model MPRs (in the reference, conventional and linear models). Values of MPR considered were 4, 6.7 (average), and 10.25. The stresses of the linear model were found to be closer to those of the reference stresses for all MPRs considered (see Fig. 9). We also observed that as the MPR of the reference configuration is varied, wall stresses considerably change.

To assess the effect of complex curvature and asymmetrical geometry on wall stresses, we considered a patient-specific geometrical AAA model with applied internal pressure (see Fig. 8C). We made the assumption that the patient AAA geometry obtained from CT scan images corresponded to the unloaded configuration; therefore, we used this geometry as the initial configuration in the reference model. Conventional and linear models used the deformed configuration obtained from the reference model (after applying an internal pressure) as their initial configuration. While stress distributions looked similar for the linear, conventional and reference models, local effective stress plots across the wall thickness (from selected regions) showed that, in general, linear stresses better approximated the reference stresses. Computed differences in stress with respect to the reference stress values (using Eq. 32) at the inner and outer surfaces, where differences in stress were expected to be larger, further revealed that stresses from the linear model, compared to those of the conventional model, were closer to the reference stresses (see Figs. 8C and S3).

### Axisymmetric Tubular Model of an AAA with Varying Tissue Properties and Residual Stresses

**Figure 10:**
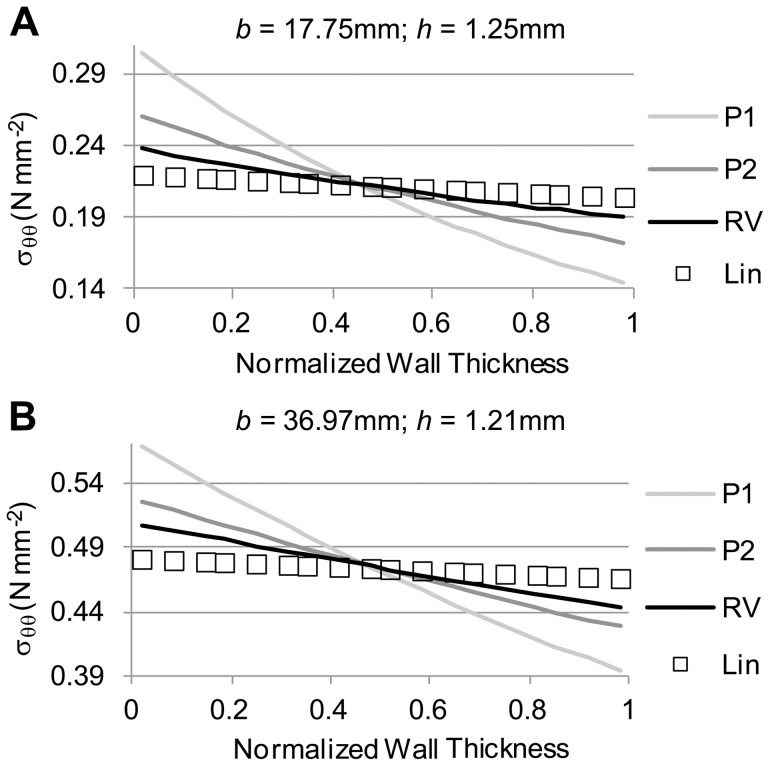
Comparison of circumferential stress distributions obtained using different tissue material properties in a tubular model. Material properties employed were those listed in Table 1 (RV, P1, and P2), and results using the linear model (Lin) are also shown for comparison. For each panel shown, regardless of the material property employed in the model the deformed configuration (described by the outer radius b and wall thickness h) was the same, while the initial, unstressed configuration was adjusted. (A) Circumferential stress distributions obtained for the case of a small aneurysm. (B) Circumferential stress distributions for a larger aneurysm model.

To determine how the choice of tissue mechanical properties affects wall stresses, we simulated the axisymmetric tubular model using different nonlinear tissue properties (RV, P1 and P2; see Table 1). For these models, the initial, unloaded configuration varied slightly, such that the deformed configuration was the same for all nonlinear models, while the linear model employed this deformed configuration as its initial geometry. Two cases were considered in which the external diameters and thicknesses of the deformed configuration were: 1) 35.5 mm and 1.25, respectively; and ii) 73.9 mm and 1.21 mm, respectively. The applied internal pressure (0.016 N/mm^2^) was the same for the two cases. We found that the wall stresses computed using the nonlinear models (RV, P1 and P2) varied significantly and were different from those computed using the linear model (see Fig. 10). As expected, however, the integral of the circumferential stresses over the deformed wall thickness, left hand side of Eq. 34, was the same for all models (since the deformed configuration was the same), indicating that equilibrium of stresses was obtained.

**Figure 11:**
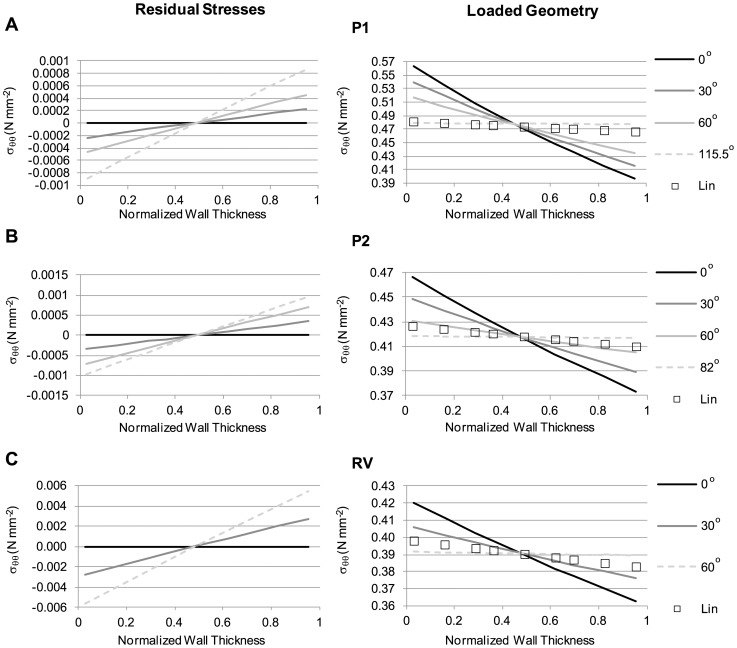
Effect of varying opening angle on circumferential residual stresses and loaded stress distributions. The model employed to compute residual stresses is schematically shown in Fig. 3. The angle *θ* was varied as indicated (with *θ = *0^°^ corresponding to the case of no residual stresses), and results obtained from employing different tissue material properties, listed in Table 1, are presented: (A) P1; (B) P2; (C) RV material properties. Results show residual stresses (left panels) in the unloaded (closed) configuration; and wall stresses after applying an internal pressure, *p* = 0.016 N/mm^2^ (right panels). Circumferential wall stresses obtained using the linear model are included for comparison. For the cases considered, the unloaded, closed configuration, which exhibits residual stresses, was the same, and characterized by *b*_0_ = 30 mm and a wall thickness of 1.5 mm. As the opening angle increased, the magnitude of residual stresses and gradient of stresses across the wall increased (right column). The magnitude of wall stresses in the loaded configuration, in contrast, decreased, and the gradient of wall stresses across the wall thickness decreased. Irrespective of the material properties employed, as *θ* increased, the magnitude of the stresses obtained using the nonlinear material properties (P1, P2, RV) approached the stress values obtained using the linear model. Dotted lines show the case at which a relatively uniform stress distribution across the wall was obtained.

Next, we considered the effect of residual stresses (see Fig. 3) on computed wall stresses. We considered cases with different material properties and different initial opening angles. We varied the angle *θ* in the initial unstressed configuration, ensuring that the closed configuration (unloaded configuration with residual stresses) was the same for all cases. In the closed configuration, the outer radius, *b*_0_, was 30 mm, while the wall thickness was 1.5 mm. An internal pressure (*p* = 0.016 N/mm^2^) was then applied to the closed configuration, and the loaded, deformed configuration was obtained. For comparison, the linear approach was applied to the deformed configuration of the case with no residual stresses (*θ* = 0°). We found that, as expected, increasing the opening angle increased the magnitude of residual stresses (see Fig. 11, left panels). Residual stresses were negative in the inner portion of the wall and positive in the outer portion of it, with magnitudes that depended on both the opening angle and wall material properties. Loading the closed geometries with an internal pressure resulted in wall stresses that were progressively smaller in magnitude with increasing opening angle (see Fig. 11, right panels). As a consequence, the gradient of stresses across the wall decreased with increasing *θ* until a flat wall stress profile across the wall thickness was obtained. Increasing the opening angle beyond this point resulted in a change in the sign of the wall stress gradient (inner wall had lower stress than the outer wall). The opening angle at which a flat wall stress profile across the wall thickness was achieved strongly depended on the wall material properties considered. Computations performed using the linear model showed a relatively flat stress profile across the wall thickness (see Fig. 11, right panels) that was representative of nonlinear models that accounted for residual stresses. These results serve as a way of elucidating possible effects of residual stresses on AAA tissue stresses, even though the residual stresses cannot currently be computed on patient-specific models.

## Discussion

Wall stress computations of vascular tissues and, in particular, of AAA tissues are difficult to achieve. This is because tissue mechanical properties are nonlinear and anisotropic and could vary spatially; cardiovascular loads generate large tissue deformations; and patient-specific geometries can be intricate while proper boundary conditions can be difficult to estimate. When stresses are computed using numerical techniques, such as FEA, nonlinearities introduce convergence problems, in which the solution does not converge to equilibrium (i.e., equilibrium of forces is not achieved). These difficulties force researchers to seek solutions by loading the tissues in small increments. These steps, however, introduce computational and model-preparation challenges that make achievement of solutions extremely difficult for non-experts and tedious for experts. Moreover, wall stresses are typically computed assuming that the loaded geometries obtained from CT scans, MRI scans, or ultrasound images are unstressed and unloaded [17] [18]. Application of internal pressures to these geometries results in large, artificial deformations and stress overestimation [22] [23], which are shown in this study. While methodologies to find the initial unloaded and unstressed configuration have been proposed [23], they are difficult to implement [18]. Moreover, current methodologies do not account for residual stresses, unknown spatial changes in material properties, or the effects of external organs on AAA tissues. Thus, computations of wall stress are extremely time-consuming and might not yet be accurate given that patient-specific tissue mechanical properties, residual stresses and outside boundary conditions are not known.

To facilitate the computation of wall stresses, we propose using linear models of AAAs. Our linear models assume not only linear wall material properties, but also infinitesimally small displacements and strains. Thus, the linear models can compute equilibrium wall stresses while preserving the loaded patient-specific geometry. The simplicity of the approach allows computations to be achieved quickly, without nonlinear iterations or small incremental load steps and without the need to know the tissue mechanical properties. We found that the proposed linear approach not only offers the benefits of computational efficiency and simplicity, but also approximates reference stresses better than conventional models in various AAA geometries. Additionally, computations using the linear model provided a desirable and physiologically relevant flat wall stress profile over the wall thickness.

### Limitations

Our linear, conventional, and reference models involved several simplifying assumptions. These assumptions included the following: i) tissue mechanical properties used were isotropic and uniform; and ii) residual stresses were generally neglected, although we included an analysis of residual stresses for idealized tubular models. These simplifications, nevertheless, are typically used in models of AAA [9] [13] [17] and therefore our study is relevant in elucidating uncertainties introduced by these assumptions.

AAA walls are best characterized as nonlinear anisotropic tissues [7] [13] [20] [26] [33] [34]. Like our study, many studies of AAA, however, have been performed assuming isotropic and uniform nonlinear mechanical properties for wall tissues [9] [13] [16] [21] [35] [36]. This is because anisotropic material properties, including the anisotropy directions, are unknown for a specific patient; are more difficult to implement than the hyperelastic isotropic material properties typically assumed; and are more prone to model convergence issues. Likewise, heterogeneities in AAA tissue properties are also not known and cannot currently be measured on patients. Similar to the conventional modeling approach with isotropic material properties, use of anisotropic and even heterogeneous material properties generates artificial model distortions. Large uncertainties are nevertheless introduced by the lack of precise knowledge of the patient-specific tissue material properties. In a recent study [21], biaxial tensile test results from anisotropic AAA tissues obtained from patients were fitted to an isotropic energy-density function with relative good correlation among tensile test data and function values. Like our study, this previous study showed that the specific choice of tissue mechanical properties employed has a large effect on wall stress, including wall stress gradients across the wall thickness. The study concluded that tissue material properties are important, and that residual stresses, which decrease stress gradients across the wall, might be needed to more accurately estimate wall stresses. Further, other studies [21] [37] [38] [39] also acknowledged that, physiologically, wall stress is likely to be nearly uniformly distributed in blood vessel walls, with residual stresses helping to achieve a more uniform stress distribution. Because equilibrium of forces is satisfied for the linear models in the intact patient geometrical configuration, and stress gradients across the wall thickness obtained using linear models of AAA are minimal, the linear approach holds promise as an effective, computationally efficient method for estimating wall stresses in patient-specific AAAs.

Even though circumferential residual stresses and longitudinal loads are present in blood vessels [37], we generally assumed the unloaded configurations to be unstressed, as done conventionally [9] [17]. We also assumed that external organs do not affect AAA wall stress. We explored, however, the effect of residual stresses on loaded tissue wall stresses. In an idealized straight tube model, residual stresses result in a more uniform circumferential stress distribution than the case with no residual stresses (see Fig. 11). A more uniform stress distribution is postulated to optimize smooth muscle performance [39] and thus it is assumed to be a more physiological scenario. This is because a uniform stress distribution also implies uniform strains (elongation) of smooth muscle cells across the wall thickness. Smooth muscle contraction efficiency is optimized when individual cells share the same strains and contract together at the same time. Residual stresses (and residual strains) therefore help to bring smooth muscle cells across the wall to a similar strain under loading conditions, which results in a more uniform mechanical environment that improves contractility [38]. AAA walls, however, have expanded and weakened through extensive remodeling, and might hold only little residual stresses and/or longitudinal stresses. This is supported by the clinical observation that AAA tissue collapses when the aneurysm is unloaded and pathology studies that demonstrate a paucity of smooth muscle cells in the wall of AAAs compared to normal aorta. Nevertheless, accurate estimations of patient-specific wall stresses might be elusive in light of large differences in wall stresses obtained using different tissue material properties from actual AAA tissue samples. Thus, even in the absence of residual stresses, the linear approach may remain effective in approximating wall stresses in AAA tissues, regardless of the limitations in our approach.

Another limitation of the linear model is its inability to capture AAA deformations throughout the cardiac cycle, which may be useful to assess wall stiffness and, perhaps, tissue mechanical changes and tissue degradation. Typically, the change in diameter of a normal aorta near the renal-aortic bifurcation throughout the cardiac cycle is about 2 mm [40]. Although AAA tissue has been reported to have less distensibility than a normal aorta due to a loss of tissue elasticity, an increase in collagen deposition, and a possible mechanical cushioning effect from the thrombus [41] [42], AAA deformations may be significant. The linear model, however, may be used together with gated imaging modalities, e.g., electrocardiography gated CT scans or MRI scans, which allow image reconstruction at specific phases of the cardiac cycle. Wall stresses specific to AAA geometries at different desired phases, e.g. end-systole and end-diastole, could then be obtained, and wall stresses can subsequently be related to the extent of deformation measured between AAA geometries. This information may be helpful in the assessment of aneurysmal tissue degradation and thus in assessments of rupture and expansion risks.

### Advantages of the Linear Model

The proposed linear model applied to AAA tissues generally yielded good approximations of wall stresses with relatively small stress gradients across the wall thickness. The wall stresses obtained with the linear model were frequently closer to reference stresses than the stresses obtained using a conventional approach. Further, the linear model captured the physiologically relevant situation of small stress gradients across the wall thickness that is a consequence of residual stresses. Because the linear model achieved equilibrium of stresses on the patient-specific geometry directly, boundary conditions (the intraluminal pressure applied to the inner AAA wall) were exactly satisfied on the deformed patient-specific geometry. This results in a reduction of artifacts due to geometrical distortions of the AAA geometry beyond those of patient tissue deformations that frequently occur when conventional approaches are used. Even when approaches that first compute the unloaded configuration are employed, in which equilibrium and boundary conditions are also satisfied directly on the patient-specific geometry, the linear approach yields wall stresses with a relatively flat stress profile across the wall. Further, these advantages are achieved in a computationally efficient way, with a relatively easy and straight-forward implementation.

Incorporation of thickness variability in models of AAA has been shown to result in significant differences in wall stress compared to models with a uniform thickness [43]. This is an important consideration because tissue thickness is typically not uniform in patients [43]. The use of uniform thickness models, however, comes from limitations in imaging technologies, from which determination of wall thickness variations is difficult. With the improvement of imaging technologies, however, it is easy to envision that wall-thickness variations would soon be incorporated into wall segmentation algorithms from images [43] [44]. The linear model could therefore be used for reliably studying the effects of wall thickness.

Advantages of the linear approach make it a promising tool for further AAA wall stress investigations and implementation in clinical practice. The linear model does not require the computation of an initial configuration; does not artificially distort the imaged, loaded geometry; and can approximate wall stresses when wall tissue properties are unknown. Further, the linear models achieve relatively small wall stress gradients across the wall thickness, which might be physiologically relevant. The linear model has the additional advantage over the conventional model (and even over models that compute the unloaded configuration) of being much faster and easier to implement, with wall stress solutions being obtained directly without the need of nonlinear iterations or time-consuming load steps. The linear approach, thus, is a robust and computationally efficient tool in computing wall stresses for patient-specific AAA studies.

### Effect of Thrombus in the Calculation of Wall Stresses

Consideration of an intraluminal thrombus (ILT) in the AAA models could be important since it decreases the magnitude of the wall stresses [45] [46]. The linear model approximated reference wall stresses very well (<5% difference) when the wall-ILT MPR were the same for the reference and linear models (see Fig. 9). This indicates that when the patient-specific wall-ILT property ratios are known, the linear approach is highly effective at estimating wall stresses. In a more clinical relevant scenario, determining the patient-specific wall-ILT MPR is not currently feasible. To circumvent this problem, we employed a mean MPR, obtained from mean patient tissue and ILT mechanical property measurements. Other groups that used conventional approaches or approaches that compute the unloaded configuration also had to rely on average tissue and ILT material properties (not only MPR). To assess uncertainties in using average properties, we employed a mean MPR value of 6.7 for the linear and conventional models, while allowing the MPR of the reference model to vary. We observed that wall stress differences between the linear and reference models vary significantly (from 3.7% to 66%, the latest for the most extreme case of MPR  = 4 in the reference model). This is, however, an intrinsic difficulty that all models face (conventional approaches yielded differences in wall stresses with respect to reference stresses that ranged from 6.3% to 111.2%) since patient-specific material properties are unknown. Thus, care will need to be exercised in the computation of wall stresses from models with thrombi to make sure that wall estimations and associated risk calculations are conservative.

### Effect of Boundary Conditions in the Calculation of Wall Stresses

Typically, continuum mechanics equations (Eqs. 1 and 2) establish equilibrium of forces in the deformed configuration. This implies that boundary conditions are applied to the final, deformed configuration. As presented before, this choice also implies that, in a cylindrical model, equilibrium in the reference and linear models will yield the same value for the integral of *σ_θθ_* over the wall thickness (see Eq. 34), ensuring that linear estimates of wall stresses are similar to reference wall stresses. The conventional nonlinear models, however, yield a different equilibrium integral because application of internal pressure produces a deformation beyond that of the imaged equilibrium configuration. Application of internal pressure boundary conditions with respect to the undeformed configuration, rather than the deformed configuration, in the conventional approach could yield stresses that are closer to those obtained using the reference model. In fact, when applying internal pressures to the undeformed configuration, the magnitude of Cauchy stresses *σ_θθ_* and *σ_rr_* were closer to reference stresses, than those obtained when the internal pressure was applied to the deformed configuration (see Figs. S4 and S5). Conventional and linear models then yielded similar estimations of wall stress. The linear model, however, not only provides and alternative way of computing AAA wall stresses, but also has the advantages of easy implementation, solution efficiency, and independence of tissue mechanical properties.

### Potential Clinical Applications

While wall stress could provide better estimation of AAA rupture risk and expansion than the maximal aneurysm diameter [5] [9] [10], current difficulties in the computation of patient-specific wall stresses, rupture risk, and AAA size progression still remain. These difficulties include uncertainties in the tissue material properties and tissue strength; computation of the unloaded configuration (including residual stresses); unknown boundary conditions (including the effect of external organs and the ILT); and the nonlinearity of the models, which increase the complexity of the computations involved. While patient-specific AAA loaded geometries can be imaged and segmented for use in FEA computation of wall stresses, these models do not account for the patient-specific tissue mechanical properties, which are unknown and challenging to obtain without tissue dissection. To circumvent these problems, researchers have been using average values of AAA tissue material properties and average tissue strengths obtained from cadaver studies or tissues obtained from patients undergoing elective repair. Thus, while stresses are calculated on patient-specific geometries and perhaps patient specific blood pressures, the remaining assumptions in the model are not patient-specific. The use of hyperelastic tissue material properties in the AAA models, in addition, makes the FEA solution difficult to achieve and time-consuming. Therefore, while several promising studies relating AAA wall stresses, AAA size progression, and rupture risk have been conducted in the research arena, these models have not been widely translated into clinical practice.

Improving the accuracy and efficiency of wall stress computations is a key step for assessing an AAA patient's risk of rupture and for improving our understanding of how wall stresses relate to AAA progression. We have shown that the use of different tissue material properties and tissue opening angles can lead to drastic changes in computed wall stresses and wall stress gradients across the wall thickness (Figs. 10 and 11). Further, wall stresses depend on the mechanical properties of the intraluminal thrombus and interaction with external organs, which are also typically unknown. The collective uncertainties introduced by unknown patient-specific tissue material properties, degree of residual stresses, and degree of tissue degradation and strength indicate that computation of truly patient-specific AAA wall stresses might be elusive. The proposed linear model provides a relatively simple methodology to estimate wall stresses, which is not only computationally efficient, but that also ensures satisfaction of wall stress equilibrium directly in the patient-specific AAA geometry. Further, the linear model does not require knowledge of tissue mechanical properties, and yields a physiologically relevant wall stress profile across the wall thickness.

Implementation of the linear model will tremendously facilitate automation of the computational process to obtain patient-specific AAA wall stresses. This can translate into the computation of patient-specific wall stresses in a much shorter time. Improving the accuracy and speed for wall stress computations are indispensable for identifying patients who are at higher risk for AAA rupture or expansion to the renal arteries or iliac bifurcation and require emergent repair. Previous studies have shown that wall stress better discriminates rupture and expansion risks than maximal AAA diameter. Studies are undergoing to determine the extent to which wall stresses determined from the linear model can indeed be used in predicting patient-specific outcomes. The proposed linear model has shown so far to be a promising clinical tool for possibly predicting AAA rupture and expansion risk. With the computation of wall stress enormously simplified by using the linear approach, studies of rupture and expansion risk can be more easily performed and extended and prediction of patient outcomes more readily obtained.

## Supporting Information

Figure S1 **Relative differences in effective wall stress distributions for the case of a tubular arterial model.** Similar to Fig. 5, differences of effective wall stress distributions obtained from the conventional and linear models, with respect to the stress distributions from the reference model are shown, but the applied internal pressure was increased to 0.027N/mm^2^ (200 mmHg). Material constants *α_ref_* and *β_ref_* reported corresponded to those of the reference model. RV material properties (*α* = 0.174 N/mm^2^, *β* = 1.881 N/mm^2^) were used in the conventional model, and constant elasticity (*E* = 8.4×10^9^ N/mm^2^) was used in the linear model. The figure shows results obtained when the initial geometry was varied in the reference model. The dashed lines indicate the physiological range of the material property values for *α_ref_*.
(TIF)

Figure S2 **Relative differences in maximum effective wall stress distributions for a tubular arterial model.** Models simulated are the same as for Fig. S1, with an applied internal pressure *p* = 0.027 N/mm^2^ (200 mmHg), but the differences in maximal wall stresses (with respect to reference wall stresses) are reported instead. Material constants *α_ref_* and *β_ref_* reported correspond to those of the reference model. RV material properties (*α* = 0.174 N/mm^2^, *β* = 1.881 N/mm^2^) were used in the conventional model, and constant elasticity (*E* = 8.4×10^9^ N/mm^2^) was used in the linear model. The figure shows results obtained when the initial geometry was varied in the reference model. The dashed lines indicate the physiological range of the material property values for *α_ref_*.
(TIF)

Figure S3 **Relative differences in effective stress on the lumen surface of a patient-specific AAA model.** Differences in effective wall stress for the linear and conventional models are with respect to the stresses in the reference model. RV material properties were employed in reference and conventional models. A systolic pressure of 0.016 N/mm^2^ (120 mmHg) was applied to the lumen of the deformed configurations of the linear and conventional models.
(TIF)

Figure S4 **Stresses in a tubular model when pressure is imposed on the conventional model's undeformed configuration.** For comparative purposes, wall stresses in the thick-wall tube are shown as obtained in the reference, conventional and linear models. Imposed internal pressure was 0.016 N/mm^2^ (120 mmHg), and was applied to the initial, undeformed configuration in the conventional model. (A) Circumferential stress; (B) effective stress; and (C) radial stress distributions are plotted across the normalized wall thickness. For the reference model, the inner and outer radii of the deformed configuration were 14.8 mm and 16.1 mm, respectively; for the conventional model, the inner and outer radii of the deformed configuration were 16.18 mm and 17.45 mm, respectively; RV material properties were used for both the reference and conventional models; *E* = 8.4×10^9^ N/mm^2^ for the linear model. Conventional models more closely approximated reference wall stresses than in the case in which pressure was applied to the deformed configuration of the conventional model.
(TIF)

Figure S5 **Patient-specific stress comparisons when internal pressure is imposed on the conventional model's undeformed configuration.** Imposed intraluminal pressure was 0.016 N/mm^2^ (120 mmHg). Effective wall stresses (in units of N/mm^2^) are shown as computed using reference, conventional and linear models (left). Differences in the effective wall stress with respect to reference wall stresses for the linear and conventional approaches are also shown (right).
(TIF)
