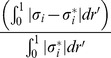# Correction: Improving the Efficiency of Abdominal Aortic Aneurysm Wall Stress Computations

**DOI:** 10.1371/journal.pone.0111567

**Published:** 2014-10-15

**Authors:** The PLOS ONE Staff

There is an error in Equation 33. Please view the complete, correct equation here: