# Supplementary figures and images for: Improving the Efficiency of Abdominal Aortic Aneurysm Wall Stress Computations
Source: PLoS One. 2014 Jul 9;9(7):e101353. doi: 10.1371/journal.pone.0101353 (PMC4090134; doi:10.1371/journal.pone.0101353)

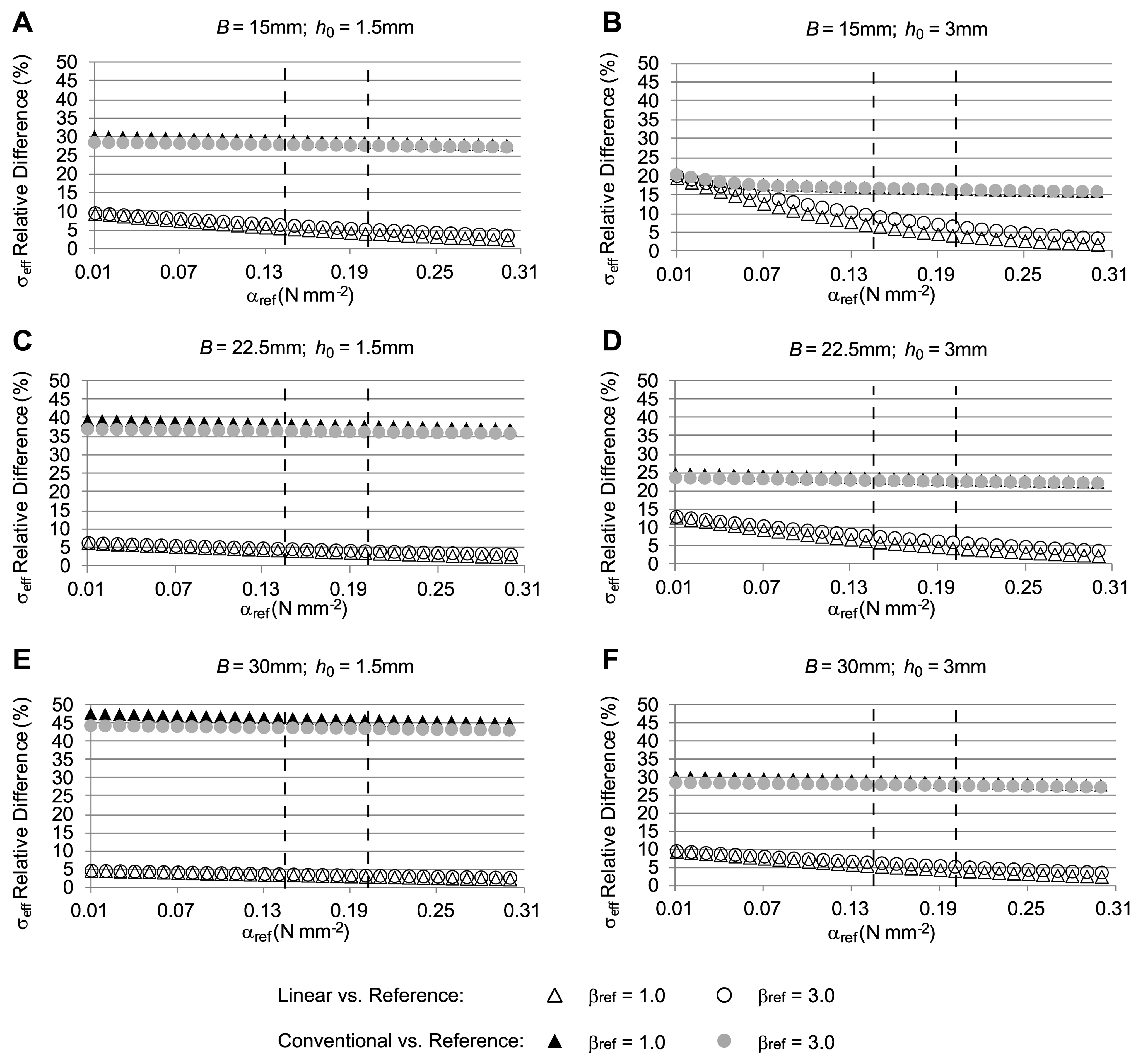

Supplement: Figure S1 — Relative differences in effective wall stress distributions for the case of a tubular arterial model. Similar to Fig. 5, differences of effective wall stress distributions obtained from the conventional and linear models, with respect to the stress distributions from the reference model are shown, but the applied internal pressure was increased to 0.027N/mm2 (200 mmHg). Material constants αref and βref reported corresponded to those of the reference model. RV material properties (α = 0.174 N/mm2, β = 1.881 N/mm2) were used in the conventional model, and constant elasticity (E = 8.4×10^9^ N/mm2) was used in the linear model. The figure shows results obtained when the initial geometry was varied in the reference model. The dashed lines indicate the physiological range of the material property values for αref. (TIF) [file pone.0101353.s001.tif]

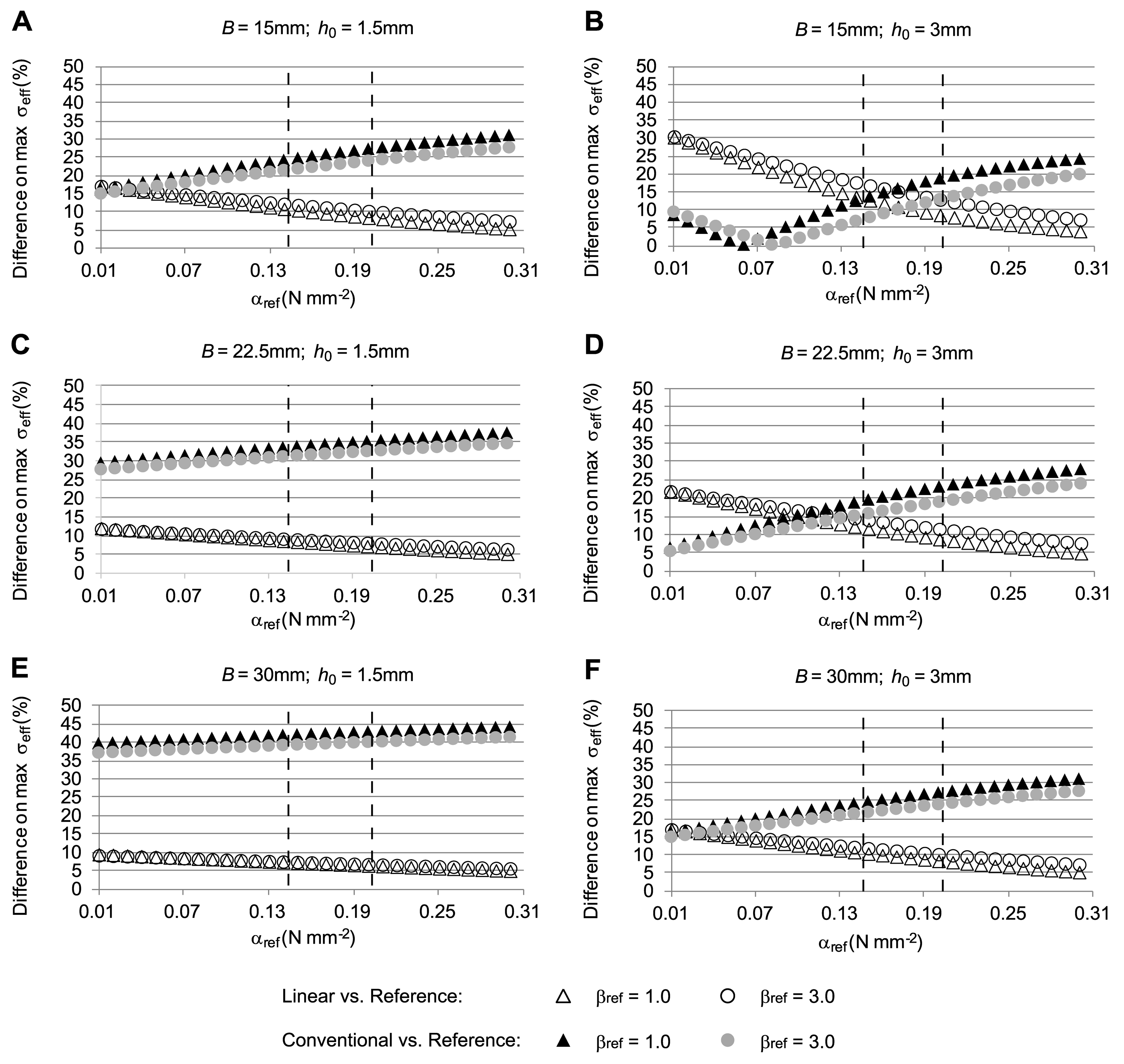

Supplement: Figure S2 — Relative differences in maximum effective wall stress distributions for a tubular arterial model. Models simulated are the same as for Fig. S1, with an applied internal pressure p = 0.027 N/mm2 (200 mmHg), but the differences in maximal wall stresses (with respect to reference wall stresses) are reported instead. Material constants αref and βref reported correspond to those of the reference model. RV material properties (α = 0.174 N/mm2, β = 1.881 N/mm2) were used in the conventional model, and constant elasticity (E = 8.4×10^9^ N/mm2) was used in the linear model. The figure shows results obtained when the initial geometry was varied in the reference model. The dashed lines indicate the physiological range of the material property values for αref. (TIF) [file pone.0101353.s002.tif]

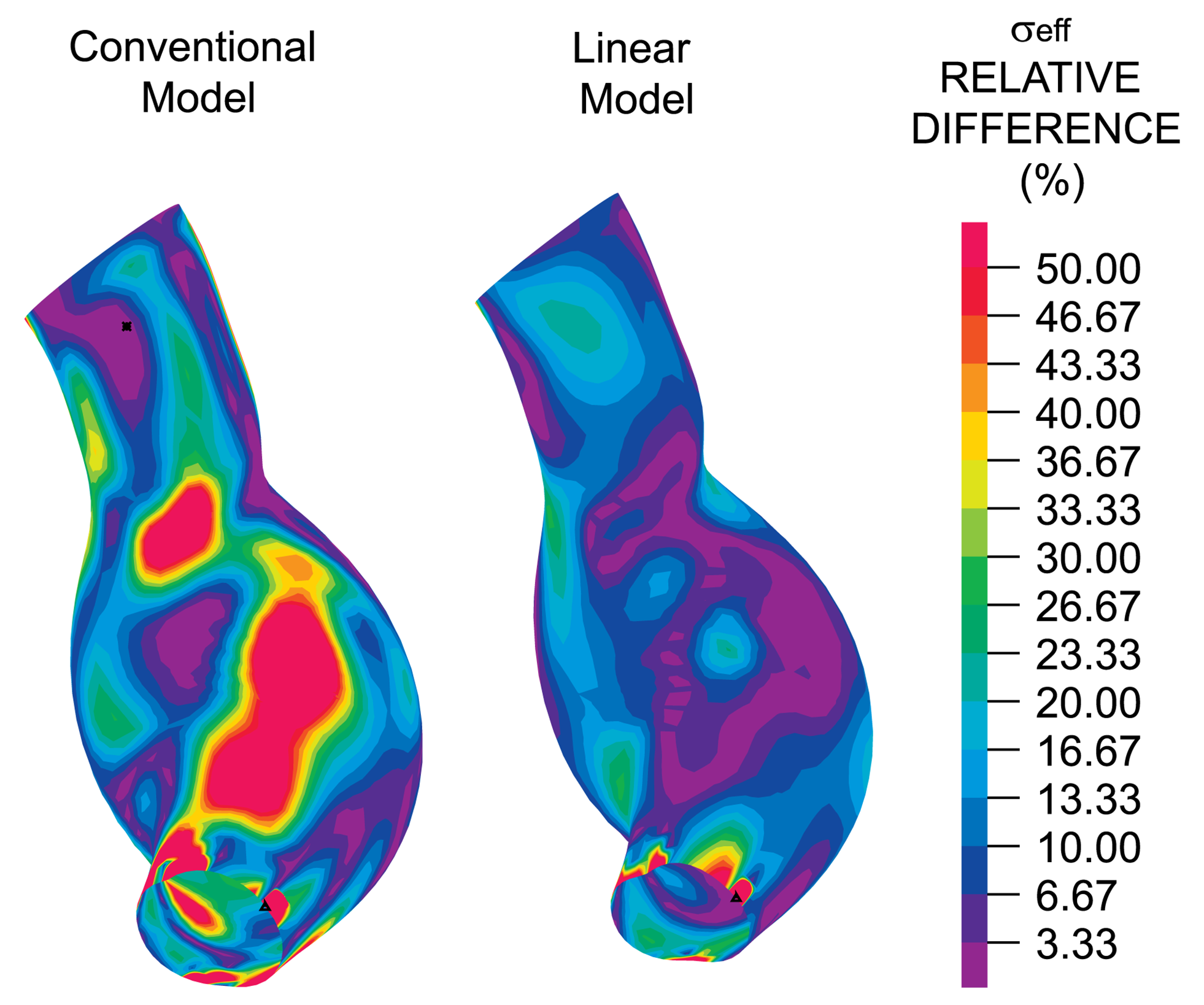

Supplement: Figure S3 — Relative differences in effective stress on the lumen surface of a patient-specific AAA model. Differences in effective wall stress for the linear and conventional models are with respect to the stresses in the reference model. RV material properties were employed in reference and conventional models. A systolic pressure of 0.016 N/mm2 (120 mmHg) was applied to the lumen of the deformed configurations of the linear and conventional models. (TIF) [file pone.0101353.s003.tif]

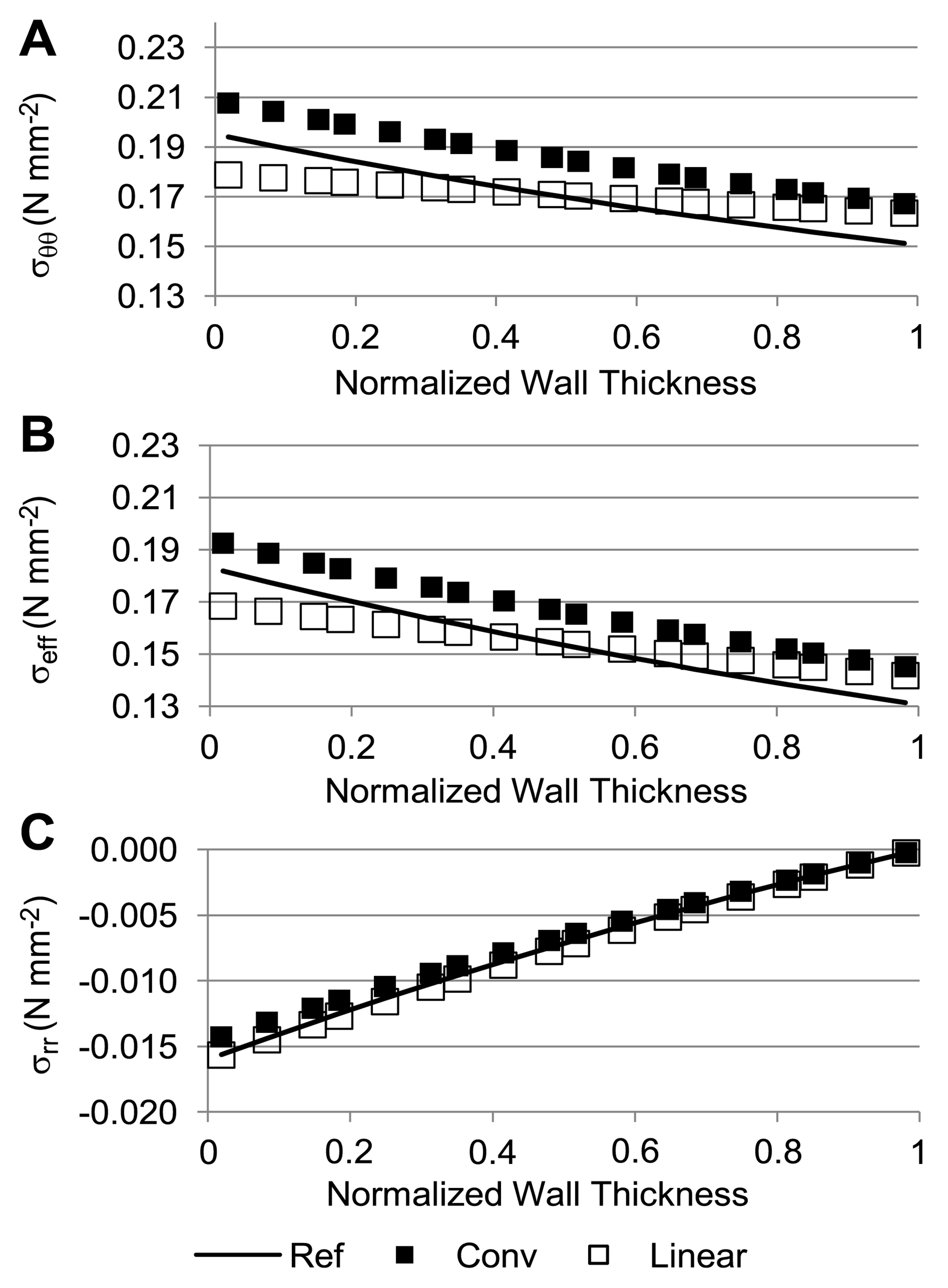

Supplement: Figure S4 — Stresses in a tubular model when pressure is imposed on the conventional model's undeformed configuration. For comparative purposes, wall stresses in the thick-wall tube are shown as obtained in the reference, conventional and linear models. Imposed internal pressure was 0.016 N/mm2 (120 mmHg), and was applied to the initial, undeformed configuration in the conventional model. (A) Circumferential stress; (B) effective stress; and (C) radial stress distributions are plotted across the normalized wall thickness. For the reference model, the inner and outer radii of the deformed configuration were 14.8 mm and 16.1 mm, respectively; for the conventional model, the inner and outer radii of the deformed configuration were 16.18 mm and 17.45 mm, respectively; RV material properties were used for both the reference and conventional models; E = 8.4×10^9^ N/mm2 for the linear model. Conventional models more closely approximated reference wall stresses than in the case in which pressure was applied to the deformed configuration of the conventional model. (TIF) [file pone.0101353.s004.tif]

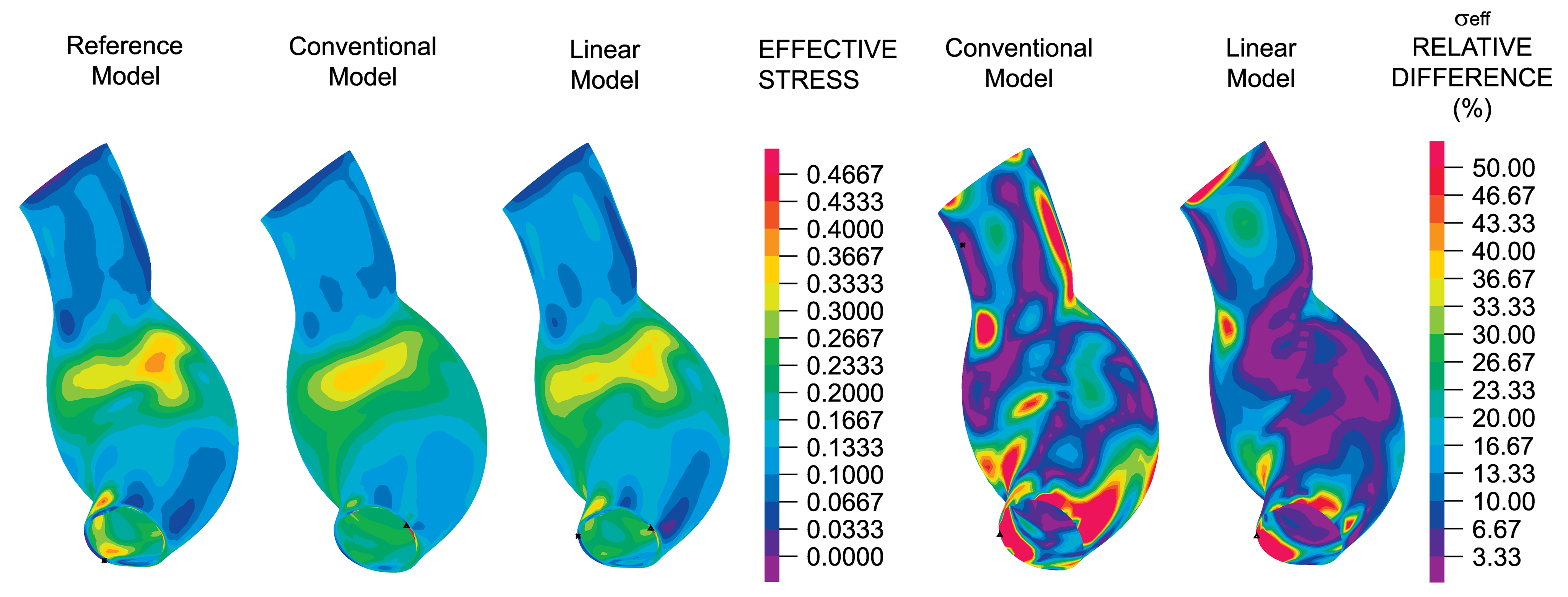

Supplement: Figure S5 — Patient-specific stress comparisons when internal pressure is imposed on the conventional model's undeformed configuration. Imposed intraluminal pressure was 0.016 N/mm2 (120 mmHg). Effective wall stresses (in units of N/mm2) are shown as computed using reference, conventional and linear models (left). Differences in the effective wall stress with respect to reference wall stresses for the linear and conventional approaches are also shown (right). (TIF) [file pone.0101353.s005.tif]
